# Analysis of Carotenoids in Haloarchaea Species from Atacama Saline Lakes by High Resolution UHPLC-Q-Orbitrap-Mass Spectrometry: Antioxidant Potential and Biological Effect on Cell Viability

**DOI:** 10.3390/antiox10081230

**Published:** 2021-07-30

**Authors:** Catherine Lizama, Javier Romero-Parra, Daniel Andrade, Felipe Riveros, Jorge Bórquez, Shakeel Ahmed, Luis Venegas-Salas, Carolina Cabalín, Mario J. Simirgiotis

**Affiliations:** 1 Departamento de Tecnología Médica, Facultad de Ciencias de la Salud, Universidad de Antofagasta, Antofagasta 1240000, Chile; catherine.lizama@uantof.cl (C.L.); daniel.andrade@uantof.cl (D.A.); felipe.riveros.c@hotmail.com (F.R.); 2 Departamento de Química Orgánica y Fisicoquímica, Facultad de Ciencias Químicas y Farmacéuticas, Universidad de Chile, Olivos 1007, Casilla 233, Santiago 6640022, Chile; javier.romero@ciq.uchile.cl; 3 Departamento de Química, Facultad de Ciencias Básicas, Universidad de Antofagasta, Antofagasta 1240000, Chile; jorge.borquez@uantof.cl; 4 Facultad de Ciencias, Instituto de Farmacia, Universidad Austral de Chile, Valdivia 5090000, Chile; shakeel.ahmed@uach.cl; 5 Departamento de Enfermedades Infecciosas e Inmunología Pediátrica, Escuela de Medicina, Pontificia Universidad Católica de Chile, Santiago 8320000, Chile; luvenegas@uc.cl (L.V.-S.); crarenas@uc.cl (C.C.)

**Keywords:** UHPLC, Orbitrap, high resolution MS analysis, haloarchaea, bacterioruberin, carotenoids, cell viability in HaCaT, Atacama saline lake, docking studies

## Abstract

Haloarchaea are extreme halophilic microorganisms belonging to the domain Archaea, phylum Euryarchaeota, and are producers of interesting antioxidant carotenoid compounds. In this study, four new strains of *Haloarcula* sp., isolated from saline lakes of the Atacama Desert, are reported and studied by high-resolution mass spectrometry (UHPLC-Q-Orbitrap-MS/MS) for the first time. In addition, determination of the carotenoid pigment profile from the new strains of *Haloarcula* sp., plus two strains of *Halorubrum tebenquichense*, and their antioxidant activity by means of several methods is reported. The effect of biomass on cellular viability in skin cell lines was also evaluated by MTT assay. The cholinesterase inhibition capacity of six haloarchaea (*Haloarcula* sp. ALT-23; *Haloarcula* sp. TeSe-41; *Haloarcula* sp. TeSe-51; *Haloarcula* sp. Te Se-89 and *Halorubrum tebenquichense* strains TeSe-85 and Te Se-86) is also reported for the first time. AChE inhibition IC_50_ was 2.96 ± 0.08 μg/mL and BuChE inhibition IC_50_ was 2.39 ± 0.09 μg/mL for the most active strain, *Halorubrum tebenquichense* Te Se-85, respectively, which is more active in BuCHe than that of the standard galantamine. Docking calculation showed that carotenoids can exert their inhibitory activity fitting into the enzyme pocket by their halves, in the presence of cholinesterase dimers.

## 1. Introduction

Haloarchaea are extreme halophilic microorganisms belonging to the domain Archaea, phylum Euryarchaeota. They are (mostly) aerobic, generally red pigmented and constitute the predominant microbial communities in extreme halophilic environments. The haloarchaea require at least 1.5 M NaCl, but most strains grow best at 3.5–4.5 M NaCl, and have been isolated from different habitats including alkaline and salt lakes, marine salterns, The Dead Sea and saline soils [1]. The Atacama Saltern is an extreme environment in the east part of the Antofagasta district of Chile and it is situated at 2300 m above sea level, at 20°30′ S and 68°15′ W. This environment has special extreme conditions that can distinguish it from other hypersaline environments. These features can be described as high salinity, high radiation (U.V), drastic changes in temperatures and high dryness. The Tebenquiche Lake is a practically anoxic [1] and hypersaline lake with neutral pH located in this region. The ionic composition of waters from this lake is dominated by sodium and chloride ions, but also with a high sulphate concentration in which the ionic dominance is Na > K > Mg > Ca = Cl > SO_4_ > HCO_3_ + CO_3_ [2]. The haloarchaea strains isolated from this special lake are a source of unexplored bioactive carotenoid compounds that protect the strains from the poly-extreme conditions of this environment. Carotenoids have been studied with great interest due to their biotechnological applications and, more importantly, their possible beneficial effects on human health [3,4]. These compounds are the second most abundant natural pigments in nature [5] and are mainly C-40 lipophilic isoprenoids ranging from colorless to yellow, orange and red [6]. The production of such types of pigments has been described from plants and some microorganisms such as algae, cyanobacteria, yeasts [7], and fungi [8]. Haloarchaea can produce carotenoids according to culture conditions, but their main reported carotenoids are composed of 50 carbon atoms. These large carotenoids are cell membrane reinforcements and protect the microorganisms against DNA damaging agents, such as salinity and solar radiation [9]. However, carotenes from halophilic archaea did not receive considerable attention [9], even though they are a main product of Atacama Desert halophilic archaea, with the 50-carbon carotenoid α-bacterioruberin being reported as their main compound [6,10,11,12,13,14]. Various studies with haloarchaea carotenoid extracts have shown potential applications in biomedicine, such as: a decrease in cell viability in human hepatoma HepG2 cells [15]; beneficial effects on the viability of sperm cells [16]; antimicrobial activity [17]; in vitro antioxidant and antihemolytic activity [18] and anticancer and antiviral activity [19]. The objective of this study is the determination of the carotenoid pigment profile of isolated haloarchaea from Tebenquiche Lake of the Atacama Desert, namely two strains of *Halorubrum tebenquichense* and four new strains of *Haloarcula* sp., isolated from this saline lake that have not been previously studied for this purpose, through high resolution mass spectrometry (UHPLC-Q-Orbitrap-MS/MS). Additionally, the total carotenoid and phenolic content is determined, and the antioxidant capacity of the carotenoid-enriched extracts verified by DPPH, FRAP and ABTS colorimetric tests. Moreover, in vitro cholinesterase tests with the extracts of the new strains were performed, plus the geometries and partial charges of every identified carotenoid optimized using semi-empirical methods and docking experiments were performed in catalytic sites of acetylcholinesterase and human butyrylcholinesterase enzymes for the first time. Cellular viability by MTT assay and the effects of the haloarchaea extract in the skin cell line HaCaT were also examined.

## 2. Materials and Methods

### 2.1. Chemicals

Ultrapure water was obtained from a water purification system brand Millipore (Milli-Q Merck Millipore, Santiago, Chile). Analytical reagents were all purchased from Sigma Aldrich Co. (Santiago, Chile). Methanol, formic acid, acetone, and acetonitrile were of chromatographic grade for HPLC analysis. Folin−Ciocalteu reagent, agarose, trolox, astaxanthin 97% by HPLC, NaOH, Na_2_CO_3_, AlCl, FeCl_3_, HCl, NaNO_2_, astaxanthin (95% purity by HPLC), trichloroacetic acid, quercetin, 6-hydroxy-2,5,7,8-tetramethylchromane-2-carboxylic acid (Trolox), sodium acetate, TPTZ, nitroblue tetrazolium, DPPH (1,1-diphenyl-2-picrylhydrazyl radical), ethanol, proteose peptone, and sodium carbonate (Na_2_CO_3_) were of analytical reagent purity and were acquired from Sigma-Aldrich Chemical Company (Santiago, Chile).

### 2.2. Strain and Cultivation Conditions

The haloarchaea strains were isolated from Tebenquiche Lake of the Atacama Saltern and belong to the collection of extreme halophilic archaea of the Laboratory of Clinical and Environmental Microbiology of the Department of Medical Technology of the University of Antofagasta. They were cultivated in medium MH containing (per litre): 5 g proteose-peptone no. 5 (Difco, Thermo Fisher, Santiago, Chile), 10 g yeast extract (Difco, Thermo Fisher, Santiago, Chile), 1 g glucose with 25% (*w*/*v*) total salts. The stock of total salts at 30% (*w*/*v*) was prepared as described by Subov (1931). Liquid cultures were grown in Erlenmeyer flasks containing 200 mL of the liquid medium and cultured to a stationary phase for 10 days with and continuous agitation at 120 rpm and 40 °C. The biomass of the haloarchaea strains was obtained by centrifugation at 10,000 rpm for 15 min, washed with a basal salt solution 20% (*w*/*v*) NaCl, and stored at −80 °C until used.

### 2.3. DNA Extraction and Amplification of 16S rRNA Encoding Gene

A 1.5 mL sample of culture the haloarchaea was harvested by centrifugation at 13,000× *g* for 5 min and 4 °C. The resulting pellet was resuspended with 1 mL distilled water. The mixture was vortexed during 1 min., and it was incubated for 60 min at 37 °C until the extract acquired a viscous texture. Then, the procedure was performed as described in [20]. PCR amplification was carried out in 50 µL volumes containing 2 mM of MgCl_2_, 2U of GoTaq G2 Flexi DNA polymerase (Promega), 150 mM of each dNTP, 0.5 mM of the primer and 2 mL of template DNA. The primers ARCH21F: TTC CGG TTG ATC CTG CCG GA [21] and 1492R: TAC GGY TAC CTT GTT ACG [22] were used for amplification of the 16S rRNA gene. The PCR program used was an initial denaturation (80 °C for 5 min), 32 cycles of denaturation (95 °C for 1 min), annealing (55 °C for 1 min) and extension (72 °C for 2 min), and a final extension (72 °C for 10 min). PCR products were visualized on a 1% agarose gel with SAFEVIEW PLUS (0.5 uL/100 mL). Sequencing was performed directly on PCR products with the universal archaeal primer sets ARCH 21F and 1492R [21,22] and Arch 344F and 915R [23] using the Macrogen sequencing service (Macrogen Inc., Gangseo-gu, Seoul, Korea).

### 2.4. Phylogenetics Analysis

Closest relatives of the 16 S rDNA sequences were determined by BLAST search (http://www.ncbi.nlm.nih.gov/blast accessed on 27 July 2021) and the classifier tool in RDP II (http://www.cme.msu.edu/rdp; accessed on 27 July 2021). Pairwise similarities between sequences were determined using p-distance in MEGA X. Trees were edited using MEGA X [24]. The sequences were deposited in GenBank for accessions for the 16S rRNA gene sequences (Supplementary Materials).

### 2.5. Preparation of the Sample for Analyses

The biomass of the haloarchaea strains was obtained by centrifugation and stored at −80 °C for two days. Then, the samples were lyophilized in a freeze-evaporation system (Model 7670541 FreeZone 2.5 Liter Labconco Freeze Dry Systems, Palo Alto, CA, USA) where all water contained in the original product was removed by freeze-evaporation cycles. The lyophilizate was stored in 15 mL Falcon centrifuge tubes protected from light, and once the dry weight was determined, it was stored at −20 °C until use. The lyophilized material (0.3–1.0 g) was added 2 mL of an acetone: water mixture in a proportion (8: 2 *v*/*v*), [25], vortexed for 10 min, then sonicated for 10 min and subsequently centrifuged at 4500 rpm for 15 min at −4 °C. Three cycles of vortexing, ultra-sounding and centrifugation were employed until a final volume of 5 mL of supernatant was recovered, which was then stored at −20 °C protected from light until use.

### 2.6. Determination of Total Phenols Content

The determination of the total phenol content (TPC) was performed by the colorimetric method of Folin–Ciocalteu with some modifications [26,27]. To 100 μL of the strain extract to be measured (at 2 mg/mL), 940 μL of Milli-Q water and 480 μL of the 10% Folin–Ciocalteu reagent (Merck, Santiago, Chile) were added in a test tube, mixed using a vortex mixer. The prepared tube was allowed to react for 5 min, and then 480 μL of 10% sodium carbonate was added. The mixture was incubated at room temperature for 30 min in darkness. Absorbance was then measured at 765 nm using a UV-visible spectrophotometer (Spectroquant Pharo 300 Merck, Santiago Chile), using as blank Milli-Q water. The obtained absorbance values were replaced in the equation of the standard curve of Trolox (curve from 10–250 μg/mL). The content of phenolic compounds was expressed as Trolox micromoles per gram of extract (μmol Trolox/g extract).

### 2.7. Determination of Total Carotenoids

The determination of the total carotenoid content (TCC) was performed according to the spectroscopy methodologies usually employed [28,29] in visible spectroscopy [30]; briefly, 90 μL of the strain extract (redissolved in acetone: water 8: 2; *v/v* at a concentration: 2 mg/mL) was added to 810 μL of the same solvent mixture and its absorbance measured at 490 nm using a 1 cm wide quartz cuvette at 25 °C in a UV-Vis spectrophotometer (Genesys 180, Thermo Fisher Scientific, San Jose, CA, USA). The total carotenoids were calculated as bacterioruberin content using its extinction coefficient (ε490) = 2600 L⋅mol^−1^⋅cm^−1^ [16], with the following formula:

$$\mathrm{Carotenoids}\;\mathrm{content}\;\mu g.g^{-1}=\frac{A\;\times \;V\;(mL)\;\times \;10^{4}}{\varepsilon 490\;\times \;P(g)}$$

where A = absorbance; V = total extract volume; and P = sample weight; ɛ490 = 2600 (Bacterioruberin extinction coefficient in acetone)

The results are expressed as micrograms per g of the extract.

### 2.8. Antioxidant Tests

#### 2.8.1. 1,1-Diphenyl-2-Picrylhydrazyl Radical Free Radical Scavenging Activity

The DPPH method previously reported by [31,32] was used to evaluate the antioxidant activity of haloarchaea strains. Briefly, 150 μL of a prepared solution of 400 μM DPPH solution was mixed with 50 μL of standards or extracts, (curve from 10–250 μg/mL). The mixture was homogenized using a vortex, allowed to react in the dark at room temperature for 20 min, after which time absorbance was measured at 517 nm. Astaxanthin was used as a reference standard (curve from 10 to 250 μg/mL). The results were expressed in Trolox micromoles per gram of extract (μmol Trolox/g extract) and as IC_50_ in μg/mL, (concentration of infusion or standard in μg/mL required to inhibit 50% of DPPH radical present in solution, as reported in [32,33]). A Synergy HTX Multi-Mode Microplate Reader (BioTek Instruments Inc., Winoosky, VT, USA) was used.

#### 2.8.2. ABTS Bleaching Capacity

The capturing capacity of the ABTS^•+^ radical was evaluated by the decolorization method developed by Re et al. (1999) and modified by Kuskoski et al. (2004) [34]. A solution of 7 mM ABTS (2,2′-azino-bis (3-ethylbenzothiazoline-6-sulphonic acid)) in deionized water and a solution of 2.45 mM potassium persulfate in water was prepared. Both solutions were mixed in a 1:1 ratio (*v*/*v*) and incubated for 16 h in the dark for the formation of the radical ABTS^•+^ by the oxidation of ABTS with potassium persulfate at room temperature. Then, to 20 μL of the extract or standard to be measured (curve from 10–250 μg/mL), 200 μL of the previously ABTS^•+^ solution (adjusted with 80% methanol to an absorbance of 0.70 ± 0.02) was added, mixed using a vortex mixer and subsequently allowed to react in darkness at room temperature [35]. The absorbance was then measured at 765 nm after 6 min of reaction, and astaxanthin was used as a reference standard (curve from 10–250 μg/mL). The results were expressed as TEAC, in Trolox micromoles per gram of extract (μmol Trolox/g extract) and also as IC_50_ in μg·mL^−1^, (concentration of infusion or standard in μg·mL^−1^ required to inhibit 50% of DPPH radical present in solution) [32,33]. A Synergy HTX Multi-Mode Microplate Reader (BioTek Instruments Inc., Winoosky, VT, USA) was used.

#### 2.8.3. Ferric Reduction Antioxidant Power Test (FRAP)

For the FRAP test, the methodology described by Benzie and Strain [32,36] was performed with some modifications. Briefly, a buffer solution made of CH_3_Nax_3_H_2_O 3.1%/CH_3_COOH (glacial) 16% dissolved in water plus 20 mM FeCl_3_ in aqueous solution HCl 0.02 M and 10 mM TPTZ dissolved in absolute ethanol was prepared. The working solution corresponded to a mixture of one volume of buffer with one volume of FeCl_3_ and 11 volumes of ethanol. The working solution (290 μL) was mixed with 10 μL of the standard Trolox (from 10 to 250 μg/mL) in a well of the microplate in quintuplicate, and a curve was prepared measuring the absorbance of the colored Fe-TPTZ complex at 593 nm after 5 min. Then, absorbance values (in quintuplicate) of strain extracts (10 μL, at a concentration: 2 μg/mL) were replaced in the Trolox standard curve equation [32,35]. The results were expressed as Trolox equivalents (TE) in Trolox micromoles per gram of extract (μmol Trolox/g extract). A Synergy HTX Multi-Mode Microplate Reader (BioTek Instruments Inc., Winoosky, VT, USA) was used.

### 2.9. Cholinesterases’ (ChE) Enzyme Inhibitory Activity

The inhibitory activity of these important enzymes was measured thorough Ellman’s method, as stated previously [32,37]. Briefly, DTNB was dissolved in buffer Tris-HCl at pH 8.0 containing 0.1 M NaCl and 0.02 M MgCl_2_. Then, a filtered sample solution dissolved in deionized water (50 µL, the final concentration of the plate ranged from 0.05 to 25 μg/mL) was mixed with 125 µL of 5-dithio-bis (2-nitrobenzoic) acid (DTNB), acetylcholinesterase (AChE), or butyrylcholinesterase (BuChE) solution (25 µL) dissolved in Tris-HCl buffer at pH 8.0 in a 96-well microplate and incubated for 15 min at 25 °C. Initiation of reaction was performed by the addition of acetyl-thiocholine iodide (ATCI) or butyryl-thiocholine chloride (BTCl) (25 µL). In addition, a blank was prepared by adding the solution sample to all reagents without the enzyme(s) (AChE or BuChE) solutions. The sample and blank absorbances were then read at 405 nm after 10 min of incubation at 25 °C. The absorbance of the sample was subtracted from that of the blank and the cholinesterase inhibitory capacity was expressed as IC_50_ (µg/mL). A Synergy HTX Multi-Mode Microplate Reader (BioTek Instruments Inc., Foster City, CA, USA) was used. Three experiments were performed in triplicate in each case and the values are reported as the mean ± SD. Galanthamine was used as positive control. 

### 2.10. UHPLC PDA-MS Instrument

A Thermo Scientific Ultimate 3000 UHPLC with PDA detector controlled by Chromeleon 7.2 Software (Thermo Fisher Scientific, Waltham, MA, USA) hyphenated with a Thermo high resolution Q-Exactive focus mass spectrometer (Thermo, Bremen, Germany) were used for analysis. The chromatographic system was coupled to the MS using a type II heated electrospray ionization source. Nitrogen obtained (purity > 99.999%) from a nitrogen generator (Genius NM32LA, Peak Scientific, Billerica, MA, USA) was employed as both the collision and damping gas. Mass calibration for Orbitrap was performed once a day, in both negative and positive modes, to ensure working mass 5 ppm of accuracy. For positive mode, a mixture of caffeine (1 mg/mL, 20 µL) and N-butylamine (1 mg/mL, 100 µL) was used, while a mixture of sodium dodecyl sulfate (1 mg/mL, 100 µL) and taurocholic acid sodium salt (1 mg/mL, 100 µL) (Sigma-Aldrich, Darmstadt, Germany) was used for negative mode. In addition, Ultramark 1621 (Alpha Aezar, Stevensville, MI, USA) was used as the reference compound (1 mg/mL, 100 µL). These compounds were dissolved in a mixture of acetic acid (100 µL), acetonitrile (5 mL), water: methanol (1:1) (5 mL) (Merck, Santiago, Chile), and 20 μL of the mixture infused using a Chemyx Fusion (Thermo Fisher Scientific, Bremen, Germany) 100 μL syringe pump and mass calibration performed every day. The strain extracts were redissolved in methanol (concentration: 2 mg/mL), filtered (PTFE filter, Merck) and 10 µL were injected in the UHPLC instrument for UHPLC-MS analysis.

### 2.11. Quantitative HPLC of Carotenoids

Liquid chromatography on an Acclaim UHPLC C-18 column (length 150 mm × 4.6 mm ID, 2.5 μm, Thermo Fisher Scientific, Bremen, Germany) as reported previously. The mobile phases were 1% formic aqueous solution (A), methanol 1% formic acid (B), and acetonitrile 1% formic acid (C). The gradient program time (min) (% B, C) was: (0.00, 18, 75); (5.00, 18, 75); (15.00, 40, 60); (20.00, 100); and 12 min for column equilibration. A curve was performed with astaxanthin (Sigma Aldrich, Santiago, Chile), covering six points, with standard solutions from 0.1 to 1 μg/mL, R^2^ = 0.9999 (LOD: 3.22 LOQ 10.43 μg/L, standard recovery rate of astaxanthin: 99.51%). The flow rate was set at 1.00 mL min^−1^, and the injection volume was 10 µL. Standards and extracts dissolved in methanol were kept at 10 °C in the autosampler. The detection wavelengths were 280, 330, 515 and 490 nm, and the diode array detector was recorded from 200 to 800 nm for peak characterization. For quantitative HPLC analysis, each sample was run in triplicate and the peak was monitored through DAD detector at 490 nm wavelength, similar to that previously reported for other extremophiles [17,38].

### 2.12. MS Parameters

The HESI II and Orbitrap spectrometer parameters were optimized as previously reported by [39]. We could use HESI instead of APCI for the fast detection of carotenoids compounds. The HESI II parameters were optimized as follows: sheath gas flow rate of 35 units; aux. gas unit flow rate of 12; capillary temperature of 320 °C; aux gas heater temperature of 500 °C; spray voltage of 3700 V (for ESI+); and S tube lens RF level of 55, as reported previously. Full scan data in both positive and negative were acquired at a resolving power of 70,000 FWHM (full width half maximum) at *m*/*z* 200. For the compounds of interest, a scan range of *m*/*z* 100–1000 was chosen; the automatic gain control (AGC) was set at 3 × 10^6^ and the injection time was set to 200 ms. Scan-rate was set at 2 scans s^−1^. External calibration was performed using a calibration solution in positive and negative modes before each sample series. In addition to the full scan acquisition method, for confirmation purposes, a targeted MS/MS analysis was performed using the mass inclusion list and expected retention times of the target analytes, with a 30 s time window, with the Orbitrap spectrometer operating both in positive and negative mode at 17,500 FWHM (*m*/*z* 200). Collision energy (HCD cell) was operated at 35 kv. Detection was based on calculated exact mass and on retention time of target compounds, presented in Table 1.

### 2.13. HaCaT Cell Culture

Immortalized human epidermal keratinocytes HaCaT cells were purchased in AddexBio catalog number #T0020001. The culture was carried out in 75 cm^2^ bottles with complete culture medium (Dulbecco’s modified Eagle medium (DMEM, Gibco Thermo fisher, Santiago, Chile) supplemented with 10% fetal bovine serum (FBS; Gibco) and 1% penicillin G-streptomycin (Gibco) at 37 °C in a humid atmosphere with 5% CO_2_. After reaching 70–80% confluence, the HaCaT cells were recovered with 0.25% trypsin-1mM EDTA and resuspended in DMEM to inactivate the trypsin. The cells were cultured again in a 96-well plate for the MTT assay.

### 2.14. Cellular Viability and Stimulation of HaCaT Cells

For this test, the biomass (strain material) of the haloarchaea was obtained by centrifugation at 10,000 rpm for 15 min from a liquid culture to 25% total salts of the medium MH. The strain *Halorubrum* *tebenquichense* Te Se-86 was selected for the testing the stimulation of HaCaT cells. The biomass obtained after centrifugation was suspended in complete culture medium for HaCaT at 1 g/mL. Then, the solution served as the stock solution to prepare serial dilutions from 1000 to 15 µg/mL. To obtain the effects of strain biomass on the cellular viability, we performed an MTT assay. For this, HaCaT cells were stimulated with different concentrations of dry biomass, obtaining a dose–response curve, including concentrations of 1000, 500, 250, 125, 62.5, 31, 15 µg/mL at different durations of stimulation of 6, 24, 48 and 72 h. The MTT assay began 2 h before the end of the stimulation, adding MTT solution dissolved in phosphate saline buffer 1× at a final concentration of 0.5 mg/mL. After 2 h, the culture medium was removed and 50 µL of dimethylsulfoxide (DMSO) was added, the solution was incubated for 10 min at 37 °C to dissolve the formazan crystals, and finally the viability was measured in a microplate reader at 545 nm. Complete medium was used in parallel as a vehicle and 10% DMSO was used as the control.

### 2.15. Oxidative Stress: Nitrite (NO_2_^−^) Concentration Assay

The Griess Reagent Kit for Nitrite Determination (Invitrogen™, Carlsbad, CA, USA) was used according to the manufacturer’s instructions to measure nitrite (NO_2_^−^), one of two primary stable and nonvolatile breakdown products of nitric oxide (NO). The procedure was performed 6 and 24 h after the end of the incubation of HaCaT cells. The level of nitrite in the cell culture medium was analyzed spectrophotometrically. The absorbance was measured at 548 nm using a multi-well plate reader (BioTek TM, Epoch, BioTek Instruments Inc., Winoosky, VT, USA). In parallel, complete medium was used as a vehicle and control.

### 2.16. Docking Assays of Carotenoids in ChE Enzymes

Docking simulations were carried for each carotenoid fragment. The geometries and partial charges of every fragment were fully optimized using the semi-empirical method for quantum calculation, Austin Model 1 (AM1 and docking calculation performed with Gaussian 09W software [40].

## 3. Results and Discussion

### 3.1. Identification of Haloarchaea Strain

**Figure 1:**
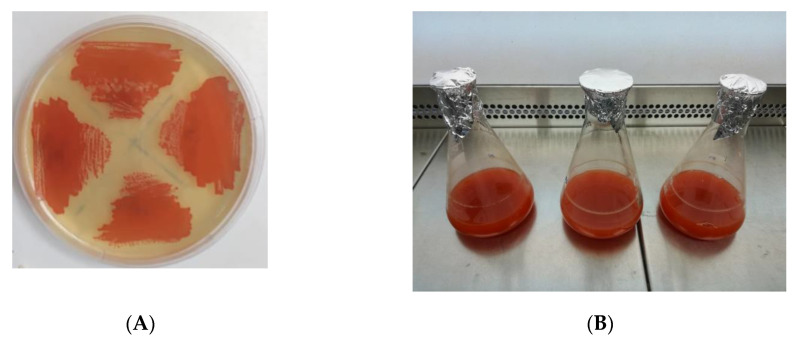
Orange-red haloarchaea colonies. (**A**) The colonies are orange in agar plates containing 25% (*w*/*v*) total salts (**B**) Culture liquid medium MH at 25% *w*/*v*, so the pigment culture changes to an intense red color after 72 h of incubation at 40 °C and 120 rpm.

**Figure 2:**
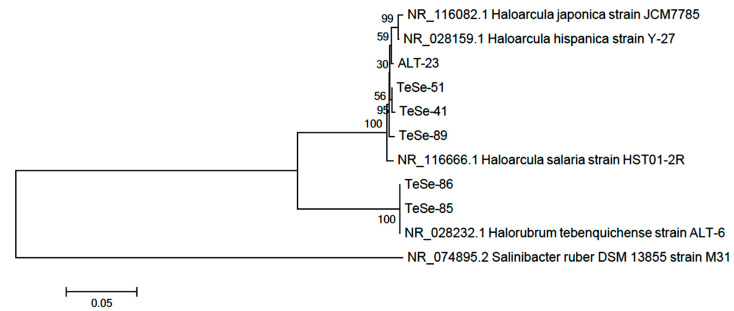
Evolutionary analyses of haloarchaea.

In this study, six new haloarchaea are reported, which have an orange-red color due to their high carotenoids content. The haloarquea strains are Gram-negative stained, non-motile and have irregular disc-shaped forms. The colonies are orange-red colored in agar plates containing 25% (*w*/*v*) total salts. In liquid medium, the pigment culture change to an intense orange-red color after 72 h of incubation (Figure 1). The analysis of the almost complete 16S rRNA gene sequence indicated that Te Se-85 and Te Se-86 strains are designated as the *Halorubrum* genus because they matched by 100% and 99.8%, respectively, with the characteristics of the reference strain *Halorubrum tebenquichense* ALT-6. The isolates ALT-23, TeSe-41, TeSe-51 are the closest to the genus *Haloarcula.* TeSe-89 strain showed 97.83% match with the identity of *Haloarcula hispanica* while the strains ALT-23; TeSe-41 and TeSe-51 showed a resemblance of 96.59%, 97.95% and 98.50% with *Haloarcula hispanica, Haloarcula salaria* and *Haloarcula japonica,* respectively [41]. The distance tree is plotted for the new sequences and the 16S rRNA sequences of other related haloarchaea (Figure 2).

The evolutionary history was inferred by using the maximum likelihood method and the general time reversible model (Figure 2) [42]. The percentage of trees in which the associated taxa clustered together is shown next to the branches. Initial trees for the heuristic search were obtained automatically by applying the neighbor-join and BioNJ algorithms to a matrix of pairwise distances estimated using the maximum composite likelihood (MCL) approach, and then selecting the topology with superior log likelihood value. The tree is drawn to scale, with branch lengths measured in the number of substitutions per site. Evolutionary analyses were conducted in MEGA X [24]. The horizontal bar at the base of the figure represents 0.05 substitutions per nucleotide site. The percentage of trees in which the associated taxa clustered together is shown next to the branches, using a bootstrap of 500.

The GenBank accessions for the 16S rRNA gene sequences are: MZ576846 (TeSe-89); MZ576847 (TeSe- 86); MZ576848 (TeSe-85); MZ576849 (TeSe-51); MZ576850 (TeSe-41) and MZ576851 (ALT-23).

### 3.2. Identification of the Carotenoids Compounds

**Figure 3:**
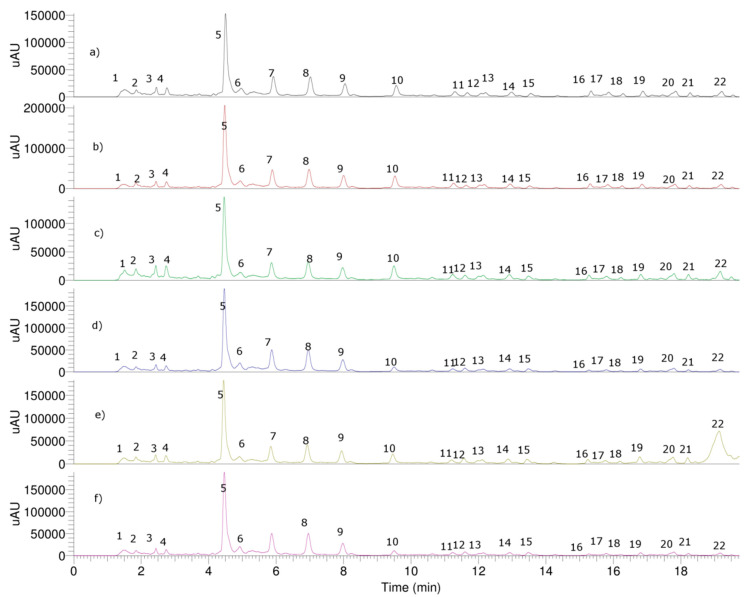
(**a**–**f**). HPLC-DAD chromatograms at 490 nm of haloarchaea strains: Te Se-85, Te Se-86, ALT-23, TeSe-41, TeSe-51 and TeSe-89.

**Figure 4:**
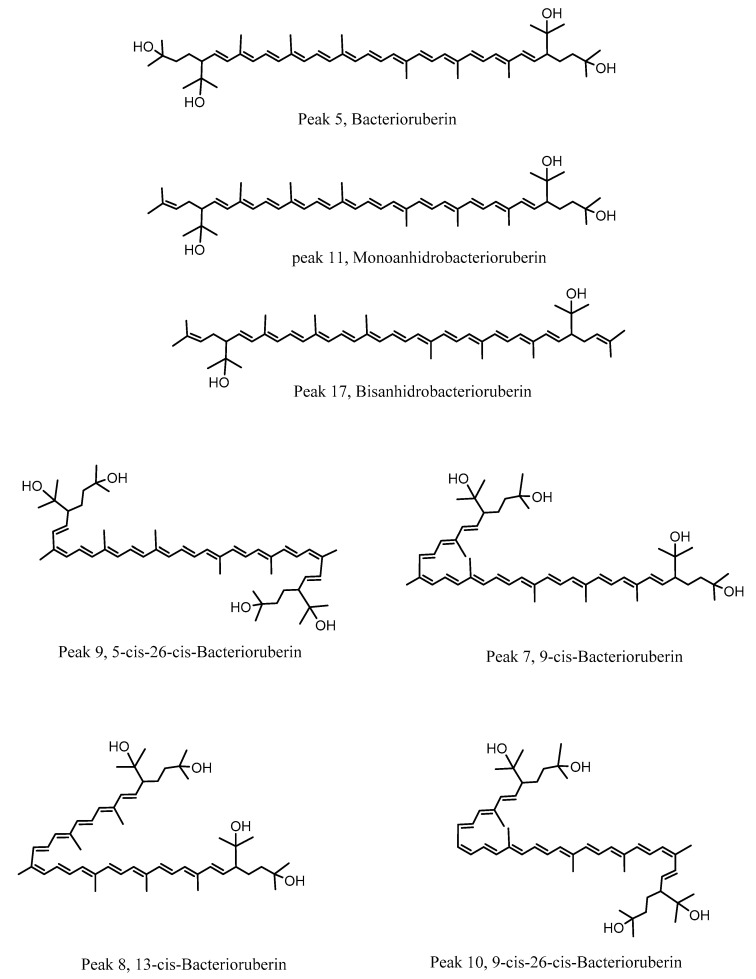
Structures of some representative carotenoids compounds found in haloarchaea.

Sixteen compounds were identified or tentatively identified by means of high resolution Orbitrap mass spectrometry (Table 1, Figure 3 and Figure 4). The fast identification of the compounds is explained below. Several compounds were identified as carotenoids, the responsible agents for the color of the strains. Peak 1 (Uv-max 318, 388, 466, 495, 528 nm) with a pseudomolecular ion at *m*/*z* 740.59998 was identified as bacterioruberin (C_50_H_74_O_4_) [43], a potent antioxidant. Peaks 6, 7 and 8 with similar molecular mass were identified as isomers of the latter with *cis* double bonds (5-*cis*-bacterioruberin, 9-*cis*-bacterioruberin and 13-*cis*-bacterioruberin) [9], respectively (Figure 3). Peaks 9 and 10 with molecular ions at *m*/*z* 740.60034 and 740.59999 were identified as 5-*cis*-26-*cis*-bacterioruberin and 9-*cis*-22-*cis*-bacterioruberin (C_50_H_74_O_4_), respectively. Peak 11 with UV max at 318, 369, 388, 465, 494, 528 nm and ion at *m*/*z* 722.58799 was identified as the dehydrated compound mono-anhidrobacterioruberin (C_50_H_74_O_3_) [44], while peaks 12, 14 and 15 were identified as the isomers of the latter, 5-*cis*-monoanhidrobacterioruberin, 9-*cis*-monoanhidrobacterioruberin and 13-*cis*-monoanhidrobacterioruberin, respectively. Peaks 13 and 16 with ions at *m*/*z* 724.60364 were identified as reduction products of peak 11 (C_50_H_76_O_3_). In the same manner, peak 17 with an ion at *m*/*z* 704.57642 was identified as the di-dehydrated carotene bisanhidrobacterioruberin (C_50_H_72_O_2_) [44], peaks 18 and 19 were identified as 5-*cis*-bisanhidrobacterioruberin and 9-*cis*-bisanhidrobacterioruberin (C_50_H_72_O_2_) [45], respectively, and finally, peak 20 with an ion at *m/z* 706.59619 was identified as reduced bisanhidrobacterioruberin.

### 3.3. Total Phenols, Carotenoids Content, Enzyme Inhibitory Activity and Antioxidant Properties of Haloarchaea

In the DPPH assays, generally, extracts for these haloarchaea showed considerable antioxidant activity [9]. The DPPH scavenging activity of our strains was shown to be similar to that of the extremophiles *Aquisalibacillus elongatus* MB592 [17] (80 percent inhibition of DPPH radical at 5–30 μg/mL). The haloarchaea *Halorubrum tebenquichense* Te Se-86 showed the highest radical trapping ability (59.14 ± 9.54 μmol Trolox/g extract, (IC_50_ = 2.95 ± 0.02 μg/mL), which is close to that exhibited by *Haloarcula hispanica* [33], followed by *Halorubrum tebenquichense* TeSe-85 (55.43 ± 3.26 μmol Trolox/g extract, IC_50_ = 4.73 ± 0.02 μg/mL); *Haloarcula* sp. TeSe-41 (41.11 ± 6.02 μmol Trolox/g extract) and *Haloarcula* sp. ALT-23 (39.84 ± 6.21 μmol Trolox/g extract). ABTS radical bleaching and reducing power FRAP assay showed the same trend, which is in accordance with the carotenoid and phenolic content found (Te Se-86 > Te Se-85 > TeSe-41 > ALT-23 > TeSe-51 > TeSe-89, Table 2). The total carotenoids compounds range from 950.2 ± 15.5 μg astaxanthin/g dry weight (Te Se-86) to 412.39 ± 19.1 μg astaxanthin/g dry weight (TeSe-89), as measured by spectrophotometry (Table 2).

Pigment carotenoids were also detected with HPLC coupled to a photodiode array detector at 490 nm wavelength (Figure 3), and the main bacterioruberin carotenoid is in *Halorubrum tebenquichense* Te Se-86 (445.0 ± 6.24 μg/g dry cells, Table 3) which also showed the higher content of total geometrical carotenoids (871.53 μg/g dry cells) measured by UHPLC. However, in terms of percentage regarding total carotenoids, bacterioruberin was higher in *Haloarcula* sp., TeSe-89 (59.5% of the total carotenoids measured by UHPLC) close to TeSe-51 (59.1%) then *Haloarcula* sp. TeSe-41 (58.2%), then *Halorubrum tebenquichense* Te Se-85 (54.6%) close to *Halorubrum tebenquichense* Te Se-86 (51.8%), followed by *Haloarcula* sp. ALT-23 (51.4%). Bacterioruberin contents in strain SGH1 from similar locations in Chile gave 427 ± 8.72 μg/g, % *w*/*v* NaCl at 25 °C [14]. However, lower carotenoid contents have been reported for other halophilic archaea; e.g., 75 µg/g of dry biomass in *Haloterrigena turkmenica* [45], 335 µg/g of dry biomass in *Haloarcula japonica* [46] and 45 µg/g of dry biomass in *Halococcus morrhuae* [12]. A bacterioruberin content of 1055.35 µg/g and total carotenoids of 2060 µg/g was reported in *Haloferax alexandrinus*, representing 51% of the total carotenoids [47], and also a huge bacterioruberin content (220 mg/g dry weight) was reported in a genetically modified *Haloferax volcanii* strain HVLON3, which also has higher antioxidant activity than standard β-carotene [16]. The application of enzyme inhibition medicines is considered effective to control several degenerative diseases, such as Alzheimer’s disease, arthritis, and arthrosis. In this study, an in vitro inhibitory effect of the dried extract (dissolved in water) of several haloarchaea, on acetylcholinesterase and butyrilcholinesterase enzymes was investigated. The results of inhibitory potencies of choline esterase inhibitors (AChE and BuChE) inhibitory activities are shown in Table 3 and expressed as IC_50_. In particular, AChE inhibition IC_50_ was 2.96 ± 0.08 mg mL^−1^ and BuChE inhibition IC_50_ was 2.39 ± 0.09 mg mL^−1^ for the most active strain, Te Se-85, respectively, (Table 2), which is more active in BuChE than that of the standard galantamine (Table 2). This is in accordance with previous results of the inhibition of these enzymes on other investigated extremophile strains (but only reported in percentage, with a maximum 40% inhibition for the AChE enzyme for the strains *Haloarcula hispanica* and *Halobacterium salinarum* for chloroform and ethyl acetate extracts of the strains) [33].

### 3.4. Effect of Biomass Haloarchaea in Skin Cell Line

**Figure 5:**
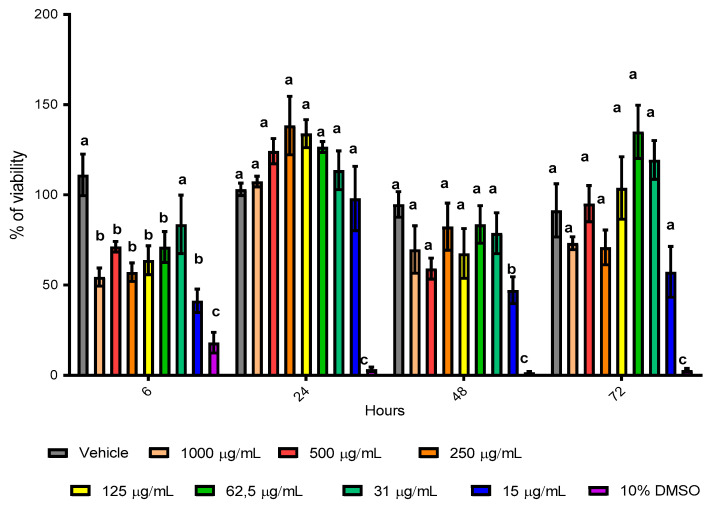
Effect of biomass in HaCaT skin cell line evaluated by MTT assay. The figure shows HaCaT cells cultivated with biomass of the strains *Halorubrum tebenquichense* Te Se-86 for 6, 24, 48 and 72 h of stimulus. The stimulus was evaluated as a percentage of viability with respect to the vehicle. Serial dilutions from 1000 to 15 µg/mL were incubated. Three independent experiments were performed. Bars indicate mean ± SD. Equal letters are not significative differences; different letters show a significative difference. *p* < 0.05, using a Kruskal–Wallis statistics test.

**Figure 6:**
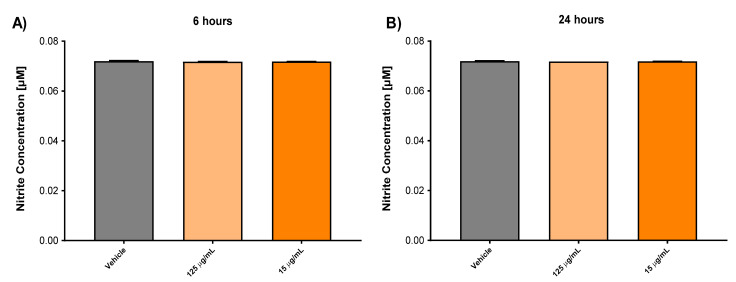
Effect of haloarchaea biomass on the secretion of nitrites in HaCaT skin cell line. The figure shows HaCaT cells cultivated with biomass of the strains *Halorubrum tebenquichense* Te Se-86 for 6 and 24 h of stimulus. The secretion of nitrites was evaluated by Griess assay, quantified as a concentration calculated in µM. (**A**) Nitrite concentration after 6 h of stimulus. (**B**) Nitrite concentration after 24 h of stimulus. Bars indicate mean ± SD, *p* < 0.05, using a Kruskal–Wallis statistics test.

The use of carotenoid antioxidant components from extremophilic microorganisms has great potential in therapies for the human health; for this reason, we decided to test the extracts from a selected haloarchaea in a skin cell line called HaCaT. Initially, the metabolic activity associated with cell viability was evaluated by the MTT assay. Cell viability in the HaCaT cell line showed a significant decrease at 6 h post stimulus at a concentration of 1000, 500, 250, 125, 62.5 µg/mL (Figure 5). However, the same concentrations do not show differences after 24, 48 and 72 h. Although the concentration of 250 µg/mL showed a trend to increase the cell viability, in the concentration of 15 µg/mL, a decrease in cell viability at 6 and 48 h was observed, while after 24 and 72 h, no significative differences were observed after cell stimulation (Figure 5). These results suggest that biomass has an effect on cellular metabolism, being able to affect the correct cellular functioning. This capacity would allow us to decrease the metabolism in cells with a high proliferation rate, such as cancer. This is what has been observed in other studies, where the use of supernatant metabolites reduces the proliferative rate of these cells, reducing the size of the tumor [48]. Moreover, the anti-cancer effect of supernatant metabolites from *Halobacterium salinarum* was published. Apoptosis was induced by overexpression of CASP3, by a reduction in pluripotency through the downregulation of SOX2 and causing the eradication of tumors in a mouse model [48]. For the same reason, to elucidate whether haloarchaea biomass influences the activation of pathways related to oxidative stress, we decided to evaluate the concentration of nitrites in the culture supernatants at 15 and 125 µg/mL at 24 and 48 h using Griess assays (Figure 6), because in the case of a possible future use, it should be tested in small concentrations. Our results do not show a significant difference in HaCaT stimulation compared to the control, showing that strain biomass does not stimulate the HaCaT cells to modify the oxidative stress response, suggesting that the decrease in viability may be due to a different mechanism. However, to understand the effect of biomass on cell repair or the inhibition of any signaling pathway, it is necessary to use in vitro or in vivo models of inflammation and cell damage [48].

### 3.5. Docking Assays of Carotenoids in Haloarchaea Species

**Figure 7:**
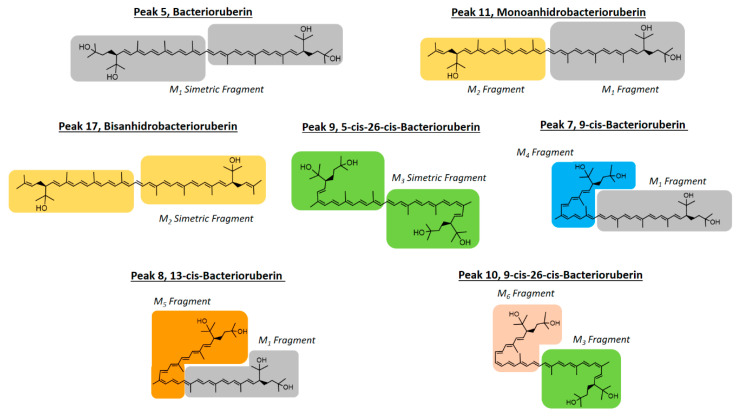
Carotenoids structures found in haloarchaea species and colored fragments tested in docking assays.

**Figure 8:**
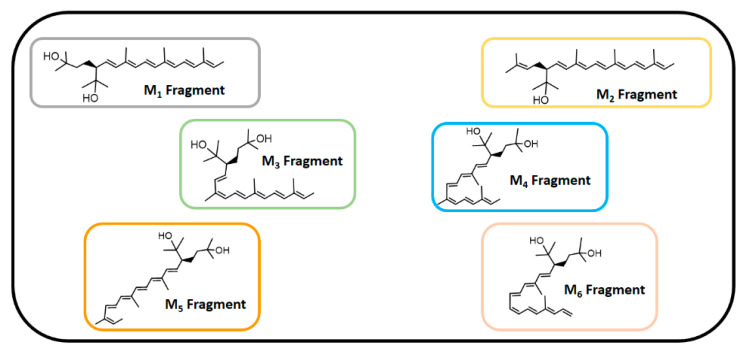
Carotenoids structure fragments used in cholinesterase docking assays.

Docking simulations were carried for each carotenoid fragment shown in Figure 7 and Figure 8 obtained from haloarchaea species. Figure 7 shows the fragmentation pattern of each carotenoid structure and Figure 8 summarizes the fragments used in cholinesterase docking assays.

#### 3.5.1. Acetylcholinesterase Docking Results

The acetylcholinesterase enzyme corresponds to a homodimer bound to the plasma membrane through covalently attached phosphatidylinositol. The monomer is an α/β protein with an ellipsoidal shape. Likewise, acetylcholinesterase catalytic site has been well established, consisting of a narrow gorge, about 20 Å long, and reaches halfway into the protein and widens out close to its bottom. The amino acids involved in the catalytic activity (known as the catalytic triad) are Ser200, Glu327 and His440, but also a hydrophobic pocket exists, where 14 residues such as Trp84, Tyr121, Trp279, Phe288, Phe290, Phe330, Phe331, Tyr334, among others, in a substantial portion of the surface of the gorge. Taking into account the above information and considering that all carotenoids shown in Figure 4 correspond to aliphatic chains of at least 50 carbon atoms, that is to say long hydrocarbon chains (30 Å length on average), they would not fit well into the acetylcholinesterase catalytic site. The latter suggest that carotenoids exert their inhibitory activity fitting into the enzyme pocket by their halves, one located half out of the catalytic site and the other performing the biological activity, or both halves can exert inhibitory activities in the case that they are in the presence of cholinesterase dimers. In this manner, we decided to fragment the carotenoid structures, since most of them show a plane of symmetry or the fragmented portions are equivalent (e.g., bacterioruberin halves-fragments are equivalent to one fragment of those exhibited by monoanhidrobacterioruberin, Figure 7).

The geometries and partial charges of every fragment were fully optimized using the semi-empirical method for quantum calculation Austin Model 1 (AM1) [49] in Gaussian 09W software [40]. Crystallographic enzyme structures of *Torpedo californica* acetylcholinesterase (*Tc*AChE; PDBID: 1DX6 code [50]) and human butyrylcholinesterase (*h*BuChE; PDBID: 4BDS code [51]) were downloaded from the Protein Data Bank RCSB PDB [52]. Docking experiments were performed using Autodock 4.2 [53], and consequently grid maps were calculated using the autogrid option and were centered on the putative catalytic site of each enzyme, considering their known catalytic amino acids: Ser200 for acetylcholinesterase (*Tc*AChE) [54,55] and Ser198 for butyrylcholinesterase (*h*BuChE) [56,57], respectively. The volumes chosen for the grid maps were made up of 60 × 60 × 60 points, with a grid-point spacing of 0.375 Å. Docked compound complexes were built using the Lamarckian genetic algorithm [58], which involved 100 runs. The lowest docked-energy binding cluster positions were chosen to be analyzed according to the potential intermolecular interactions between inhibitors and the enzymes. The different complexes were displayed in a visual molecular dynamics program (VMD) and Pymol [59].

Butyrylcholinesterase belongs to the same structural class of proteins as acetylcholinesterase, belonging to the esterase/lipase family, and being a serine hydrolase as well [60,61]. The butyrylcholinesterase catalytic triad is composed of Ser198, Glu325 and His438. The catalytic site, such as acetylcholinesterase, is located at the bottom of a deep and narrow gorge of the enzyme. One of the main differences between butyrylcholinesterase and acetylcholinesterase are some residues lining the gorge, where the former enzyme has replaced several of the aromatic groups of the latter by hydrophobic ones [62]. In view of the information already mentioned, we also decided to use the carotenoid halves main fragments depicted in Figure 8 to perform docking assays over butyrylcholinesterase.

Table 4 shows the best docking binding energies expressed in kcal/mol of each carotenoid fragments (M_1_, M_2_, M_3_, M_4_, M_5_ and M_6_) obtained from haloarchaea species, as well as the known cholinesterase inhibitor galantamine. Docking results would allow us to rationalize the carotenoid inhibitory properties through the analysis of the proteins molecular interactions in the light of the obtained results shown in Table 2.

**Figure 9:**
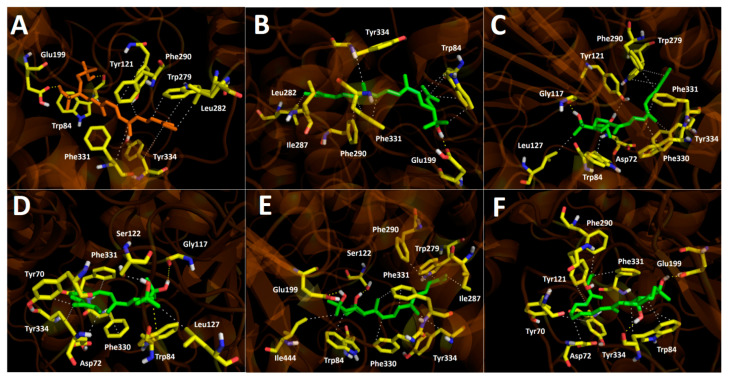
Predicted binding mode and predicted intermolecular interactions of different fragments (Figure 8) in the catalytic site of acetylcholinesterase; (**A**) fragment M_1_ in the catalytic site (**B**) fragment M_2_ in the catalytic site; (**C**) fragment M_3_ in the catalytic site; (**D**) fragment M_4_ in the catalytic site; (**E**) fragment M_5_ in the catalytic site; (**F**) fragment M_6_ in the catalytic site.

All fragments displayed several hydrophobic interactions in the acetylcholinesterase catalytic site, specifically with the hydrophobic residues at the surface of the catalytic gorge, which probably represents the main components of their inhibitory activities. In addition, the hydroxyl groups located in the fragments demonstrated to produce hydrogen bond interactions with amino acids Trp82, Tyr121, Gly117, Ser122 and Glu199 as well (Figure 9A–F).

Fragment M_1_, which constitutes part of the structures of bacterioruberin, monoanhidrobacterioruberin, 9-*cis*-bacterioruberin and 13-*cis*-bacterioruberin, exhibited a binding energy value of −9.74 kcal/mol. Fragment M_5_ showed the worst binding energy and is present only in 13-*cis*-bacterioruberin structure. Additionally, 13-*cis*-bacterioruberin contains fragment M_1_, which, as can be seen in Table 4, did not possess the best binding energy value. Thus, it is probable that 13-*cis*-bacterioruberin would not be the most potent carotenoid as an acetylcholinesterase inhibitor.

Fragment M_2_, contained in monoanhidrobacterioruberin and bisanhidrobacterioruberin, showed a good binding energy and one hydrogen bond interaction and plenty of hydrophobic interactions with amino acid residues, including Trp 84, Leu282, Ile287 and Phe 290, among others. This suggests that both carotenoids mentioned above would behave as acetylcholinesterase inhibitors as well; nonetheless, as bisanhidrobacterioruberin corresponds to a symmetric structure containing two portions of fragment M_2_, it is possible that this derivative would demonstrate a better inhibitory profile over the enzyme.

Fragment M_3_ displayed the best binding energy, and it is the only fragment that arranges in a different manner into the acetylcholinesterase catalytic pocket projecting its hydroxyl groups toward a different direction, performing hydrogen bond interactions with the residues Tyr121 and Gly117 (Figure 9C). The different fitting of fragment M_3_ related to the other derivatives could be attributed to the cis double bond near to the branched chains that bear the hydroxyl groups. Fragment M_3_ can be found exclusively in the symmetrical 5-*cis*-26-*cis*-bacterioruberin carotenoid, which suggests that this derivative could exhibit the highest potency feature compared to other carotenoids. 9-*cis*-26-*cis*-bacterioruberin bears fragment M_3_ as well, but also contains fragment M_6_ in its structure, which raises the possibility that both fragments of 9-*cis*-26-*cis*-bacterioruberin could exert the inhibitory activity in the acetylcholinesterase catalytic site, competing between each other. What is mentioned above could harm the inhibitory profile of this derivative, considering that fragment M_6_ presented a binding energy of −9.54 kcal/mol, lower than fragment M_3_ and similar to fragment M_4_, since the former fragment (M_6_) only differs in a methyl group from the latter fragment (M_4_) in their structures (Figure 8).

In view of docking results, the best carotenoid fragment turned out to be fragment M_3_, and the worst fragment was M_5_, which is only borne by the 13-*cis*-bacterioruberin derivative. Nonetheless, all of them showed a similar manner of being arranged into the catalytic site, and hydrophobic interactions were shown to be an important non-competitive intermolecular binding docking descriptor.

#### 3.5.2. Butyrylcholinesterase Docking Results

Binding energies from docking assays over butyrylcholinesterase of fragments M_1_, M_2_, M_3_, M_4_, M_5_ and M_6_ showed a similar behavior pattern in terms of binding energies regarding those shown by acetylcholinesterase. No substantial differences among themselves or in comparison with the known inhibitor galantamine were found. These features can be corroborated through the comparison of the half-maximal inhibitory concentrations (IC_50_) presented by the six different haloarchaea obtained from the II region of Chile (Table 2).

**Figure 10:**
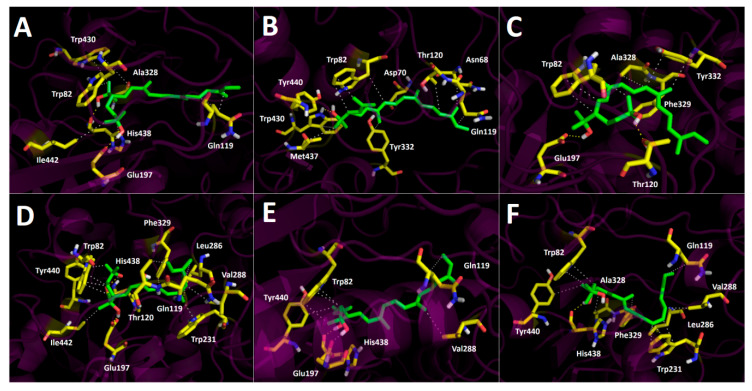
Predicted binding mode and predicted intermolecular interactions of different fragments (Figure 8) in the catalytic site of butyrylcholinestesase; (**A**) fragment M_1_ in the catalytic site; (**B**) fragment M_2_ in the catalytic site; (**C**) fragment M_3_ in the catalytic site; (**D**) fragment M_4_ in the catalytic site; (**E**) fragment M_5_ in the catalytic site; (**F**) fragment M_6_ in the catalytic site.

As in acetylcholinesterase docking assays, hydrophobic interactions also predominated in all fragments, with the residues Trp82, Gln 119, Phe 329, Trp430, among others, being implicated (Figure 10A–F). All fragments were arranged into the butyrylcholinesterase catalytic site in a similar manner, except for fragment M_3_ again, probably due to its cis double bond near to the branched chains that bear the hydroxyl groups, projecting them in different directions and leading to the obtention of hydrogen bond interactions with amino acids Glu197 and Thr120 (Figure 10C).

Fragment M_1_ showed the lowest binding energy profile, suggesting that all carotenoids which bear this fragment, such as bacterioruberin, monoanhidrobacterioruberin, 9-*cis*-bacterioruberin and 13-*cis*-bacterioruberin could mainly exert their inhibitory activities through the other fragment that each of them also possesses, except carotenoid bacterioruberin, since it is a symmetrical molecule bearing two fragment M_1_ portions.

9-*cis*-bacterioruberin represents a special case, due to it being sectionated in two different fragments (fragment M_1_ and fragment M_4_). Since, 9-*cis*-bacterioruberin bears fragment M_4_, it is possible that if an isolated inhibition assay over butyrylcholinesterase of this derivative were performed, the main activity could be attributed to fragment M_4_ instead of fragment M_1_, considering the high binding energy of −9.40 kcal/mol exhibited by this fragment, and hence a better binding affinity of it. Something similar happens with derivative 13-*cis*-bacterioruberin sectionated in fragment M_1_ and fragment M_5_ (Figure 7), where the whole inhibitory activity could fall over fragment M_5_.

Monoanhidrobacterioruberin possesses in its structure fragment M_1_ and fragment M_2_. However, both fragments demonstrated low binding energies, it which suggests that this carotenoid would not behave as the most potent inhibitor. On the other hand, bisanhidrobacterioruberin is a symmetrical compound bearing fragment M_2_ twice, but as in monoanhidrobacterioruberin, fragment M_2_ also exhibits a low binding energy profile.

Fragment M_6_ showed the best binding energy (−9.90 kcal/mol), and the only carotenoid that contains this fragment corresponds to 9-*cis*-26-*cis*-bacterioruberin. However, 9-*cis*-26-*cis*-bacterioruberin also possesses fragment M_3_ (Figure 7), due the ability of the former fragment to fit into the butyrylcholinesterase catalytic site, turning this derivative into a good candidate to become a potent butyrylcholinesterase inhibitor.

## 4. Conclusions

Six haloarchaea strains from the extreme environment of the Atacama Desert were identified, namely *Halorubrum tebenquichense* Te Se-85, *Halorubrum tebenquichense* Te Se-86, *Haloarcula* sp. ALT-23, *Haloarcula* sp. TeSe-41, *Haloarcula* sp. TeSe-51 and *Haloarcula* sp. TeSe-89. Antioxidant and cholinesterase enzymes inhibitory capacities supported by docking experiments plus carotene content were measured, and the presence of sixteen geometric bacterioruberin carotenoids was identified in the six haloarchaea. The compounds were identified by ultrahigh resolution liquid chromatography orbitrap MS analysis (UHPLC-PDA-OT-MS), and are reported for the first time, updating the knowledge on the chemical profile of these species. The effect of biomass on cellular viability in skin cell lines was also evaluated by MTT assay. More scientific data on bioactivity and chemistry are shown for these extremophiles that increase their potential for sustainable applications and industrial purposes. These valuable natural products biomass has great potential applications in food, medicine, biotechnology, pharmaceuticals, and cosmetics, with many possible applications from food coloring agents to anticancer biomaterials.

## Figures and Tables

**Table 1:** UHPLC-Q-OT-MS/MS data of haloarchaea strains: Te Se-85, Te Se-86, ALT-23, TeSe-41, TeSe-51 and TeSe-89. Peak numbers refer to Figure 3.

Peak #	Rt (min.)	λ max (nm)	Tentative Identification	Molecular Formula	Theoretical Mass (*m*/*z*)	Measured Mass (*m*/*z*)	Accuracy (δppm)	MS^n^ ions (*m*/*z*)
1	2.75		Unknown	-		156.04250		
2	2.75		Unknown	-		220.11290		
3	2.44		Unknown	-		394.29160		
4	2.75		Possibly glucosyl bacterioruberin derivative	C_55_H_83_O_8_		871.61510		420.30792
5	4.45	318, 388, 466, 495, 528	Bacterioruberin	C_50_H_74_O_4_	740.57436	740.59998	34.59	665.54810, 681.12321
6	4.92	387, 466, 492, 525	5-*cis*-Bacterioruberin	C_50_H_74_O_4_	740.57436	740.60022	34.91	443.30157, 723.32542,
7	5.91	387, 466, 492, 525	9-*cis*-Bacterioruberin	C_50_H_74_O_4_	740.57436	740.60052	35.32	682.55681,577.12548
8	6.31	387, 466, 488, 525	13-*cis*-Bacterioruberin	C_50_H_74_O_4_	740.57436	740.59991	34.50	682.55310
9	7.02	387, 466, 488, 525	5-*cis*-26-cis-Bacterioruberin	C_50_H_74_O_4_	740.57436	740.60034	34.50	
10	8.04	387, 466, 488, 525	9-*cis*-26-cis-Bacterioruberin	C_50_H_74_O_4_	740.57436	740.599991	34.60	
11	9.52	318, 369, 388, 465, 494, 528	Monoanhidrobacterioruberin	C_50_H_74_O_3_	722.56680	722.58799	29.94	
12	11.26	369, 385, 465, 492, 524	5-*cis*-Monoanhidrobacterioruberin	C_50_H_74_O_3_	722.56680	722.58820	29.61	
13	11.61	318, 369, 388, 459, 484, 516	Reduced monoanhidrobacterioruberin	C_50_H_76_O_3_	724.57945	724.60364	33.38	
14	12.09	318, 369, 388, 465, 495, 528	9-*cis*-monoanhidrobacterioruberin	C_50_H_74_O_3_	722.56680	722.58856	30.11	
15	12.94	318, 369, 388, 465, 495, 528	13-*cis*-monoanhidrobacterioruberin	C_50_H_74_O_3_	722.56680	722.58826	29.69	
16	13.51	340,377, 458,483,515	Reduced monoanhidrobacterioruberin	C_50_H_76_O_3_	724.57945	724.60413	34.06	
17	15.31	370, 389, 460, 494, 527	Bisanhidrobacterioruberin	C_50_H_72_O_2_	704.55323	704.57642	32.91	
18	15.84	370, 386, 460, 491, 524	5-*cis*-Bisanhidrobacterioruberin	C_50_H_72_O_2_	704.55323	704.57617	32.55	
19	16.26	370, 386, 464, 489, 524	9-*cis*-Bisanhidrobacterioruberin	C_50_H_72_O_2_	704.55323	704.57660	33.16	
20	16.86	370, 388,459, 485, 517	Reduced Bisanhidrobacterioruberin	C_50_H_74_O_2_	706.56888	706.59619	38.65	
21	17.83	17.83	Unknown	-	-	475.44403		
22			Unknown	-	-	708.60803		

**#**: number MS^n^: fragment ions.

**Table 2:** Scavenging of the 1,1-diphenyl-2-picrylhydrazyl radical (DPPH), radical ABTS, (ABTS), total phenolic content (TPC), total carotenoid content (TCC) and cholinesterase inhibition capacity of six haloarchaea (Te Se-85, Te Se-86, ALT-23, TeSe-41, TeSe-51 and TeSe-89) from the II Region of Chile. (*n* = 5).

Sample	DPPH ^a^	ABTS ^b^	FRAP ^c^	TPC ^d^	TCC ^e^	ACHe ^f^	BuCHe ^f^
Te Se-85	55.43 ± 3.26 ^i^ (IC_50_ = 4.73 ± 0.02 μg/mL)	473.50 ± 15.85 (IC_50_ = 7.98 ± 0.03 μg/mL)	878.07 ± 11.60	175.62 ± 8.26	714.4 ± 11.5	2.96 ± 0.08	2.39 ± 0.09
Te Se-86	59.14 ± 9.54 ^i^ (IC_50_ = 2.95 ± 0.02 μg/mL)	570.54 ± 15.45 (IC_50_ = 4.23 ± 0.02 μg/mL)	1023.03 ± 10.23	199.32 ± 7.54	950.2 ± 15.5	4.24 ± 0.10	8.63 ± 0.06
ALT-23	39.84 ± 6.21 ^h^ (IC_50_ = 8.83 ± 0.03 μg/mL)	342.46 ± 10.95 (IC_50_ = 12.12 ± 0.04 μg/mL)	669.26 ± 8.89	136.63 ± 5.22	522.05 ± 18.7 ^k,l^	7.89 ± 0.04	18.33 ± 0.07
TeSe-41	41.11 ± 6.02 ^h^ (IC_50_ = 6.25 ± 0.01 μg/mL)	376.38 ± 12.06 (IC_50_ = 9.32 ± 0.02 μg/mL)	762.50 ± 10.64	146.19 ± 6.27	540.7 ± 12.2 ^l^	2.57 ± 0.04	7.57 ± 0.05
TeSe-51	26.13 ± 3.25 ^g^ (IC_50_ = 15.43 ± 0.04 μg/mL)	272.61 ± 11.31 ^j^ (IC_50_ = 18.45 ± 0.08 μg/mL)	479.27 ± 9.37	98.64 ± 6.35	512.63 ± 13.5 ^k^	7.80 ± 0.03	3.52 ± 0.04
TeSe-89	23.10 ± 4.68 ^g^ (IC_50_ = 23.19 ± 0.04 μg/mL)	250.66 ± 18.70 ^j^ (IC_50_ = 34.72 ± 0.06 μg/mL)	396.56 ± 21.11	79.85 ± 5.48	412.39 ± 19.1	3.04 ± 0.05	17.83 ± 0.07
Astaxanthin	63.15 ± 6.25 (IC_50_ = 7.22 ± 0.02 μg/mL)	585.73 ± 13.22 (IC_50_ = 89.47 ± 0.08 μg/mL)	1045.56 ± 13.43	212.73 ± 8.83	923.45 ± 20.3	-	-
Galantamine						0.26 ± 0.02	3.82 ± 0.02

^a^ Antiradical DPPH activities are expressed as μmol Trolox/g extract; ^b,c^ expressed as μmol Trolox/g extract; ^d^ total phenolic content (TPC) expressed as μmol Trolox/g dry weight; ^e^ total carotenoid content (TCC) expressed as μg astaxanthin/g dry weight. ^f^ CHe enzyme e inhibitory activity in IC_50_ in μg/mL. Values in the same column marked with the same letter are not significantly different (at *p* < 0.05).

**Table 3:** Total bacterioruberin carotenoids in haloarchaea: Te Se-85, Te Se-86, ALT-23, TeSe-41, TeSe-51 and TeSe-89 by UHPLC analyses.

Strain	Haloarchaea Content (mg/g Dry Biomass)					
	Te Se-85	Te Se-86	ALT-23	TeSe-41	TeSe-51	TeSe-89
Total carotenoids *	600.06	871.53	488.88	536.93	532.89	508.412
Bacterioruberin	328.9 ± 3.23	445.0 ± 6.24	251.52 ± 4.22	312.31 ± 3.02	315.92 ± 3.13	303.03 ± 2.86
5-*cis*-Bacterioruberin	27.84 ± 1.45	45.12 ± 1.36	18.87 ± 0.98	26.65 ± 1.22	25.13 ± 1.06	28.32 ± 1.18
9-*ci*s-Bacterioruberin	52.43 ± 1.86	83.59 ± 1.75	41.36 ± 1.22	50.12 ± 1.38	46.44 ± 1.32	55.232 ± 1.48
13-*cis*-Bacterioruberin	56.98 ± 0.98	88.23 ± 1.45	49.26 ± 0.65	48.45 ± 1.66	54.87 ± 1.22	50.16 ± 1.26
5-*cis*-26-*cis*-Bacterioruberin	43.54 ± 1.43	76.44 ± 1.95	39.12 ± 1.22	32.66 ± 1.75	43.26 ± 1.34	37.76 ± 1.08
9-*cis-*26-*cis*-Bacterioruberin	22.35 ± 0.96	33.54 ± 1.01	21.87 ± 0.56	15.33 ± 1.25	20.14 ± 1.12	11.15 ± 0.24
Monoanhidrobacterioruberin	9.22 ± 0.54	15.22 ± 0.65	9.21 ± 0.46	7.23 ± 0.43	6.32 ± 0.35	5.78 ± 0.36
5-*cis*-monoanhidrobacterioruberin	7.25 ± 0.46	12.26 ± 0.57	6.31 ± 0.37	5.32 ± 0.32	4.28 ± 0.16	3.66 ± 0.26
Reduced monoanhidrobacterioruberin	5.12 ± 0.21	8.32 ± 0.23	5.12 ± 0.21	3.46 ± 0.27	2.38 ± 0.21	1.87 ± 0.17
9-*cis-*Monoanhidrobacterioruberin	6.34 ± 0.31	9.47 ± 0.42	5.32 ± 0.16	5.59 ± 0.18	1.98 ± 0.17	0.87 ± 0.12
13-*cis*-Monoanhidrobacterioruberin	6.28 ± 0.28	9.16 ± 0.67	4.39 ± 0.19	5.93 ± 0.43	2.25 ± 0.15	0.69 ± 0.17
Reduced Monoanhidrobacterioruberin	7.32 ± 0.25	9.46 ± 0.33	5.26 ± 0.23	5.67 ± 0.48	1.83 ± 0.03	0.91 ± 0.05
Bisanhidrobacterioruberin	5.03 ± 0.11	8.16 ± 0.12	5.26 ± 0.22	3.92 ± 0.34	1.78 ± 0.31	1.95 ± 0.26
5-*cis*-bisanhidrobacterioruberin	4.81 ± 0.24	7.42 ± 0.15	4.11 ± 0.13	2.55 ± 0.23	0.67 ± 0.21	0.73 ± 0.12
9-*cis-*bisanhidrobacterioruberin	7.23 ± 0.34	9.25 ± 0.33	9.45 ± 0.45	4.76 ± 0.23	2.23 ± 0.45	2.43 ± 0.37
Reduced Bisanhidrobacterioruberin	9.42 ± 0.45	10.89 ± 0.47	12.45 ± 0.74	6.98 ± 0.74	3.41 ± 0.36	3.87 ± 0.27

* Sum of individual carotenoids compounds.

**Table 4:** Binding energies obtained from docking experiments of carotenoids in haloarchaea species over acetylcholinesterase and butyrylcholinesterase.

Compound	Binding Energy (kcal/mol) Acetylcholinesterase	Binding Energy (kcal/mol) Butyrylcholinesterase
Fragment M_1_	−9.74	−7.42
Fragment M_2_	−10.15	−7.89
Fragment M_3_	−10.35	−8.35
Fragment M_4_	−9.53	−9.40
Fragment M_5_	−8.50	−8.62
Fragment M_6_	−9.54	−9.90
Galantamine	−11.81	−9.50

## Data Availability

The data presented in this study are available in the article and its Supplementary Materials.

## Supplementary Materials

The following are available online at: https://www.mdpi.com/article/10.3390/antiox10081230/s1. Figure S1: UV visible spectra of bacterioruberins from haloarchaea. Figures S2 and S3: Full orbitrap MS spectra and structures of some representative compounds. Register of the 16S rRNA gene sequences.

## References

[1] Lizama C., Monteoliva-Sánchez M., Prado B., Ramos-Cormenzana A., Weckesser J., Campos V. (2001). Taxonomic study of extreme halophilic archaea isolated from the “Salar de Atacama”, Chile. Syst. Appl. Microbiol..

[2] Farias M.E., Rasuk M.C., Gallagher K.L., Contreras M., Kurth D., Fernandez A.B., Poiré D., Novoa F., Visscher P.T. (2017). Prokaryotic diversity and biogeochemical characteristics of benthic microbial ecosystems at La Brava, a hypersaline lake at Salar de Atacama, Chile. PLoS ONE.

[3] Zhang J., Sun Z., Sun P., Chen T., Chen F. (2014). Microalgal carotenoids: Beneficial effects and potential in human health. Food Funct..

[4] Vílchez C., Forján E., Cuaresma M., Bédmar F., Garbayo I., Vega J.M. (2011). Marine carotenoids: Biological functions and commercial applications. Mar. Drugs.

[5] Sun T., Yuan H., Cao H., Yazdani M., Tadmor Y., Li L. (2018). Carotenoid Metabolism in Plants: The Role of Plastids. Mol. Plant.

[6] Yatsunami R., Ando A., Yang Y., Takaichi S., Kohno M., Matsumura Y., Ikeda H., Fukui T., Nakasone K., Fujita N. (2014). Identification of carotenoids from the extremely halophilic archaeon *Haloarcula japonica*. Front. Microbiol..

[7] Mata-Gómez L.C., Montañez J.C., Méndez-Zavala A., Aguilar C.N. (2014). Biotechnological production of carotenoids by yeasts: An overview. Microb. Cell Fact..

[8] Dufossé L., Fouillaud M., Caro Y., Mapari S.A.S., Sutthiwong N. (2014). Filamentous fungi are large-scale producers of pigments and colorants for the food industry. Curr. Opin. Biotechnol..

[9] Calegari-Santos R., Diogo R.A., Fontana J.D., Bonfim T.M.B. (2016). Carotenoid Production by *Halophilic Archaea* under Different Culture Conditions. Curr. Microbiol..

[10] Jehlička J., Edwards H.G.M., Oren A. (2013). Bacterioruberin and salinixanthin carotenoids of extremely halophilic *Archaea* and *Bacteria*: A Raman spectroscopic study. Spectrochim. Acta Part A Mol. Biomol. Spectrosc..

[11] Naziri D., Hamidi M., Hassanzadeh S., Tarhriz V., Zanjani B.M., Nazemyieh H., Hejazi M.A., Hejazi M.S. (2014). Analysis of carotenoid production by *Halorubrum* sp. TBZ126; an extremely halophilic archeon from Urmia Lake. Adv. Pharm. Bull..

[12] Mandelli F., Miranda V.S., Rodrigues E., Mercadante A.Z. (2012). Identification of carotenoids with high antioxidant capacity produced by extremophile microorganisms. World J. Microbiol. Biotechnol..

[13] De la Vega M., Sayago A., Ariza J., Barneto A.G., León R. (2016). Characterization of a bacterioruberin-producing *Haloarchaea* isolated from the marshlands of the Odiel river in the southwest of Spain. Biotechnol. Prog..

[14] Flores N., Hoyos S., Venegas M., Galetović A., Zúñiga L.M., Fábrega F., Paredes B., Salazar-Ardiles C., Vilo C., Ascaso C. (2020). *Haloterrigena* sp. Strain SGH1, a Bacterioruberin-Rich, Perchlorate-Tolerant Halophilic Archaeon Isolated From Halite Microbial Communities, Atacama Desert, Chile. Front. Microbiol..

[15] Abbes M., Baati H., Guermazi S., Messina C., Santulli A., Gharsallah N., Ammar E. (2013). Biological properties of carotenoids extracted from *Halobacterium halobium* isolated from a Tunisian solar saltern. BMC Complement. Altern. Med..

[16] Zalazar L., Pagola P., Miró M.V., Churio M.S., Cerletti M., Martínez C., Iniesta-Cuerda M., Soler A.J., Cesari A., De Castro R. (2019). Bacterioruberin extracts from a genetically modified hyperpigmented *Haloferax volcanii* strain: Antioxidant activity and bioactive properties on sperm cells. J. Appl. Microbiol..

[17] Fariq A., Yasmin A., Jamil M. (2019). Production, characterization and antimicrobial activities of bio-pigments by *Aquisalibacillus elongatus* MB592, *Salinicoccus sesuvii* MB597, and *Halomonas aquamarina* MB598 isolated from Khewra Salt Range, Pakistan. Extremophiles.

[18] Hou J., Cui H.L. (2018). In Vitro Antioxidant, Antihemolytic, and Anticancer Activity of the Carotenoids from *Halophilic Archaea*. Curr. Microbiol..

[19] Hegazy G.E., Abu-Serie M.M., Abo-Elela G.M., Ghozlan H., Sabry S.A., Soliman N.A., Abdel-Fattah Y.R. (2020). In vitro dual (anticancer and antiviral) activity of the carotenoids produced by haloalkaliphilic archaeon *Natrialba* sp. M6. Sci. Rep..

[20] Urdiain M., López-López A., Gonzalo C., Busse H.J., Langer S., Kämpfer P., Rosselló-Móra R. (2008). Reclassification of *Rhodobium marinum* and *Rhodobium pfennigii* as *Afifella marina* gen. nov. comb. nov. and *Afifella pfennigii* comb. nov., a new genus of photoheterotrophic Alphaproteobacteria and emended descriptions of *Rhodobium*, *Rhodobium orientis* and *Rhodobium gokarnense*. Syst. Appl. Microbiol..

[21] Delong E.F. (1992). *Archaea* in coastal marine environments (achabaterla/phyoey/batwe nt/n a eclogU). Proc. Natl. Acad. Sci. USA.

[22] Weisburg W.G., Barns S.M., Pelletier D.A., Lane D.J. (1991). 16S ribosomal DNA amplification for phylogenetic study. J. Bacteriol..

[23] Stahl A.D. (1991). Nucleic Acid Techniques in Bacterial Systematics. Dev. Appl. Nucleic Acid Probes.

[24] Kumar S., Stecher G., Li M., Knyaz C., Tamura K. (2018). MEGA X: Molecular evolutionary genetics analysis across computing platforms. Mol. Biol. Evol..

[25] Giani M., Martínez-Espinosa R.M. (2020). Carotenoids as a Protection Mechanism against Oxidative Stress in *Haloferax mediterranei*. Antioxidants.

[26] Simirgiotis M.J., Schmeda-Hirschmann G., Bórquez J., Kennelly E.J. (2013). The *Passiflora tripartita* (banana passion) fruit: A source of bioactive flavonoid C-glycosides isolated by HSCCC and characterized by HPLC-DAD-ESI/MS/MS. Molecules.

[27] Brito A., Areche C., Sepúlveda B., Kennelly E.J., Simirgiotis M.J. (2014). Anthocyanin characterization, total phenolic quantification and antioxidant features of some chilean edible berry extracts. Molecules.

[28] Lichtenthaler H.K., Wellburn A.R. (1983). Determinations of total carotenoids and chlorophylls a and b of leaf extracts in different solvents. Biochem. Soc. Trans..

[29] Aguilera Á., Suominen S., Pétursdóttir S., Olgudóttir E., Guðmundsdóttir E.E., Altamirano M., González-Toril E., Hreggviðsson G.Ó. (2020). Physiological plasticity of high-temperature intertidal cyanobacterial microbial mats to temperature and salinity: Daily and seasonal in situ photosynthetic performance. Eur. J. Phycol..

[30] De Carvalho L.M.J., Gomes P.B., de Oliveira Godoy R.L., Pacheco S., do Monte P.H.F., de Carvalho J.L.V., Nutti M.R., Neves A.C.L., Vieira A.C.R.A., Ramos S.R.R. (2012). Total carotenoid content, α-carotene and β-carotene, of landrace pumpkins (*Cucurbita moschata* Duch): A preliminary study. Food Res. Int..

[31] Zeraik M.L., Queiroz E.F., Marcourt L., Ciclet O., Castro-Gamboa I., Silva D.H.S., Cuendet M., da Silva Bolzani V., Wolfender J.-L. (2016). Antioxidants, quinone reductase inducers and acetylcholinesterase inhibitors from *Spondias tuberosa* fruits. J. Funct. Foods.

[32] Barrientos R., Fernández-Galleguillos C., Pastene E., Simirgiotis M., Romero-Parra J., Ahmed S., Echeverría J. (2020). Metabolomic Analysis, Fast Isolation of Phenolic Compounds, and Evaluation of Biological Activities of the Bark From *Weinmannia trichosperma* Cav. (*Cunoniaceae*). Front. Pharmacol..

[33] Gómez-Villegas P., Vigara J., Vila M., Varela J., Barreira L., Léon R. (2020). Antioxidant, antimicrobial, and bioactive potential of two new haloarchaeal strains isolated from odiel salterns (Southwest Spain). Biology.

[34] Kuskoski E.M., Asuero A.G., García-Parilla M.C., Troncoso A.M., Fett R. (2004). Actividad antioxidante de pigmentos antociánicos. Ciênc. Tecnol. Aliment..

[35] Areche C., Hernandez M., Cano T., Ticona J., Cortes C., Simirgiotis M., Caceres F., Borquez J., Echeverría J., Sepulveda B. (2020). *Corryocactus brevistylus* (K. Schum. ex Vaupel) Britton & Rose (*Cactaceae*): Antioxidant, Gastroprotective Effects, and Metabolomic Profiling by Ultrahigh-Pressure Liquid Chromatography and Electrospray High Resolution Orbitrap Tandem Mass Spectrometry. Front. Pharmacol..

[36] Benzie I.F.F., Strain J.J. (1996). The Ferric Reducing Ability of Plasma (FRAP) as a Measure of “Antioxidant Power”: The FRAP Assay. Anal. Biochem..

[37] Mocan A., Zengin G., Simirgiotis M., Schafberg M., Mollica A., Vodnar D.C., Crişan G., Rohn S. (2017). Functional constituents of wild and cultivated Goji (*L. barbarum* L.) leaves: Phytochemical characterization, biological profile, and computational studies. J. Enzyme Inhib. Med. Chem..

[38] Keerthi S., Koduru U.D., Nittala S.S., Parine N.R. (2018). The heterotrophic eubacterial and archaeal co-inhabitants of the halophilic *Dunaliella salina* in solar salterns fed by Bay of Bengal along south eastern coast of India. Saudi J. Biol. Sci..

[39] Balasubramaniam V., June Chelyn L., Vimala S., Mohd Fairulnizal M.N., Brownlee I.A., Amin I. (2020). Carotenoid composition and antioxidant potential of *Eucheuma denticulatum*, *Sargassum polycystum* and *Caulerpa lentillifera*. Heliyon.

[40] Frisch A. (2009). Gaussian 09W Reference.

[41] BLAST: Basic Local Alignment Search Tool. https://blast.ncbi.nlm.nih.gov/Blast.cgi.

[42] Nei M., Kumar S. (2000). Molecular Evolution and Phylogenetics.

[43] Sahli K., Gomri M.A., Esclapez J., Gómez-Villegas P., Ghennai O., Bonete M.-J., León R., Kharroub K. (2020). Bioprospecting and characterization of pigmented halophilic archaeal strains from Algerian hypersaline environments with analysis of carotenoids produced by *Halorubrum* sp. BS2. J. Basic Microbiol..

[44] Rodrigo-Baños M., Garbayo I., Vílchez C., Bonete M.J., Martínez-Espinosa R.M. (2015). Carotenoids from *Haloarchaea* and their potential in biotechnology. Mar. Drugs.

[45] Squillaci G., Parrella R., Carbone V., Minasi P., La Cara F., Morana A. (2017). Carotenoids from the extreme halophilic archaeon *Haloterrigena turkmenica*: Identification and antioxidant activity. Extremophiles.

[46] Yang Y., Yatsunami R., Ando A., Miyoko N., Fukui T., Takaichi S., Nakamura S. (2015). Complete biosynthetic pathway of the C50 carotenoid bacterioruberin from lycopene in the extremely halophilic archaeon *Haloarcula japonica*. J. Bacteriol..

[47] Asker D., Awad T., Ohta Y. (2002). Lipids of *Haloferax alexandrinus* strain TMT: An extremely halophilic canthaxanthin-producing archaeon. J. Biosci. Bioeng..

[48] Safarpour A., Ebrahimi M., Fazeli S.A.S., Amoozegar M.A. (2019). Supernatant metabolites from *Halophilic Archaea* to reduce tumorigenesis in prostate cancer in-vitro and in-vivo. Iran. J. Pharm. Res..

[49] Dewar M.J.S., Zoebisch E.G., Healy E.F., Stewart J.J.P. (1985). AM1: A New General Purpose Quantum Mechanical Molecular Model1. J. Am. Chem. Soc..

[50] Greenblatt H.M., Kryger G., Lewis T., Silman I., Sussman J.L. (1999). Structure of acetylcholinesterase complexed with (-)-galanthamine at 2.3 Å resolution. FEBS Lett..

[51] Nachon F., Ehret-Sabatier L., Loew D., Colas C., Van Dorsselaer A., Goeldner M. (1998). Trp82 and Tyr332 are involved in two quaternary ammonium binding domains of human butyrylcholinesterase as revealed by photo affinity labeling with [3H]DDF. Biochemistry.

[52] Berman H.M., Westbrook J., Feng Z., Gilliland G., Bhat T.N., Weissig H., Shindyalov I.N., Bourne P.E. (2000). The Protein Data Bank. Nucleic Acids Res..

[53] Morris G.M., Ruth H., Lindstrom W., Sanner M.F., Belew R.K., Goodsell D.S., Olson A.J. (2009). Software news and updates AutoDock4 and AutoDockTools4: Automated docking with selective receptor flexibility. J. Comput. Chem..

[54] Silman I., Harel M., Axelsen P., Raves M., Sussman J.L. (1994). Three-dimensional structures of acetylcholinesterase and of its complexes with anticholinesterase agents. Biochemical Society Transactions.

[55] Sussman J.L., Harel M., Frolow F., Oefner C., Goldman A., Toker L., Silman I. (1991). Atomic structure of acetylcholinesterase from Torpedo californica: A prototypic acetylcholine-binding protein. Science.

[56] Nicolet Y., Lockridge O., Masson P., Fontecilla-Camps J.C., Nachon F. (2003). Crystal Structure of Human Butyrylcholinesterase and of Its Complexes with Substrate and Products. J. Biol. Chem..

[57] Tallini L.R., Bastida J., Cortes N., Osorio E.H., Theoduloz C., Schmeda-Hirschmann G. (2018). Cholinesterase inhibition activity, alkaloid profiling and molecular docking of chilean rhodophiala (*Amaryllidaceae*). Molecules.

[58] Thomsen R., Christensen M.H. (2006). MolDock: A new technique for high-accuracy molecular docking. J. Med. Chem..

[59] PyMOL|pymol.org. https://pymol.org/2/.

[60] Kovarik Z., Radić Z., Berman H.A., Simeon-Rudolf V., Reiner E., Taylor P. (2003). Acetylcholinesterase active centre and gorge conformations analysed by combinatorial mutations and enantiomeric phosphonates. Biochem. J..

[61] Chatonnet A., Lockridge O. (1989). Comparison of butyrylcholinesterase and acetylcholinesterase. Biochem. J..

[62] Howes M.-J.R., Perry N.S.L., Houghton P.J. (2003). Plants with traditional uses and activities, relevant to the management of Alzheimer’s disease and other cognitive disorders. Phyther. Res..

