# Supplementary material for: Analysis of Carotenoids in Haloarchaea Species from Atacama Saline Lakes by High Resolution UHPLC-Q-Orbitrap-Mass Spectrometry: Antioxidant Potential and Biological Effect on Cell Viability
Source: Antioxidants (Basel). 2021 Jul 30;10(8):1230. doi: 10.3390/antiox10081230 (PMC8389013; doi:10.3390/antiox10081230)
Supplement: Supplementary file 1 [file antioxidants-10-01230-s001.zip › antioxidants-1309699-supplementary.pdf]

Supplementary material

Article

Analysis of Carotenoids in Haloarchaea Species from Atacama Saline Lakes by High Resolution UHPLC-Q-Orbitrap-Mass Spectrometry: Antioxidant Potential and Biological Effect on Cell Viability

Catherine Lizama [1,*], Javier Romero Parra [2], Daniel Andrade [1], Felipe Riveros [1], Jorge Bórquez [3], Shakeel Ahmed [4], Luis Venegas-Salas [5], Carolina Cabalín [5] and Mario J. Simirgiotis[4,*]

1. Departamento de Tecnología Médica, Facultad de Ciencias de la Salud, Universidad de Antofagasta, Chile; felipe.riveros.c@hotmail.com (F.R), daniel.andrade.bq@uantof.cl (D.A).
2. Departamento de Química Orgánica y Fisicoquímica, Facultad de Ciencias Químicas y Farmacéuticas, Universidad de Chile, Olivos 1007, Casilla 233, Santiago, Chile, javier.romero@ciq.uchile.cl
3. Departamento de Química, Facultad de Ciencias Básicas, Universidad de Antofagasta, Chile; jorge.borquez@uantof.cl. (J.B).
4. Instituto de Farmacia, Facultad de Ciencias, Universidad Austral de Chile, Valdivia, Chile; shakeel.ahmed@uach.cl (S:A)
5. Departamento de Enfermedades Infecciosas e Inmunología Pediátrica, Escuela de Medicina, Pontificia Universidad Católica de Chile, Santiago, Chile, luvenegas@uc.cl (L.V), crarenas@uc.cl (C.C)

* Correspondence: catherine.lizama@uantof.cl; mario.simirgiotis@uach.cl; Tel.: +56 988377059 (C.L.); +56 999835427 (M.S)

Figure S1. General UV visible spectra of ruberins from Haloarchaeas.

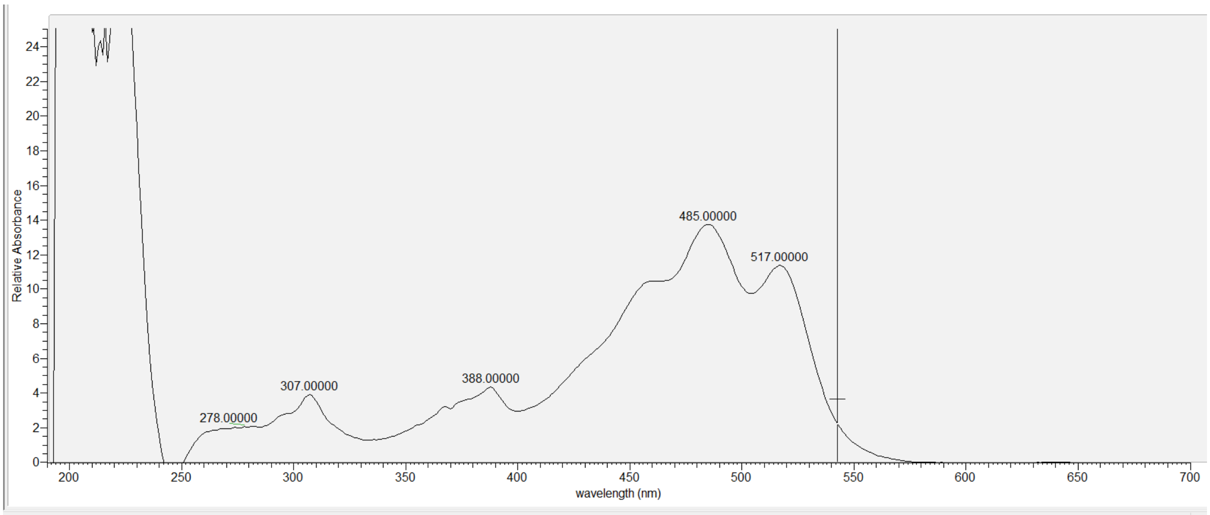

Figure S2. Full MS spectra of Bacterioruberin, peak 5.

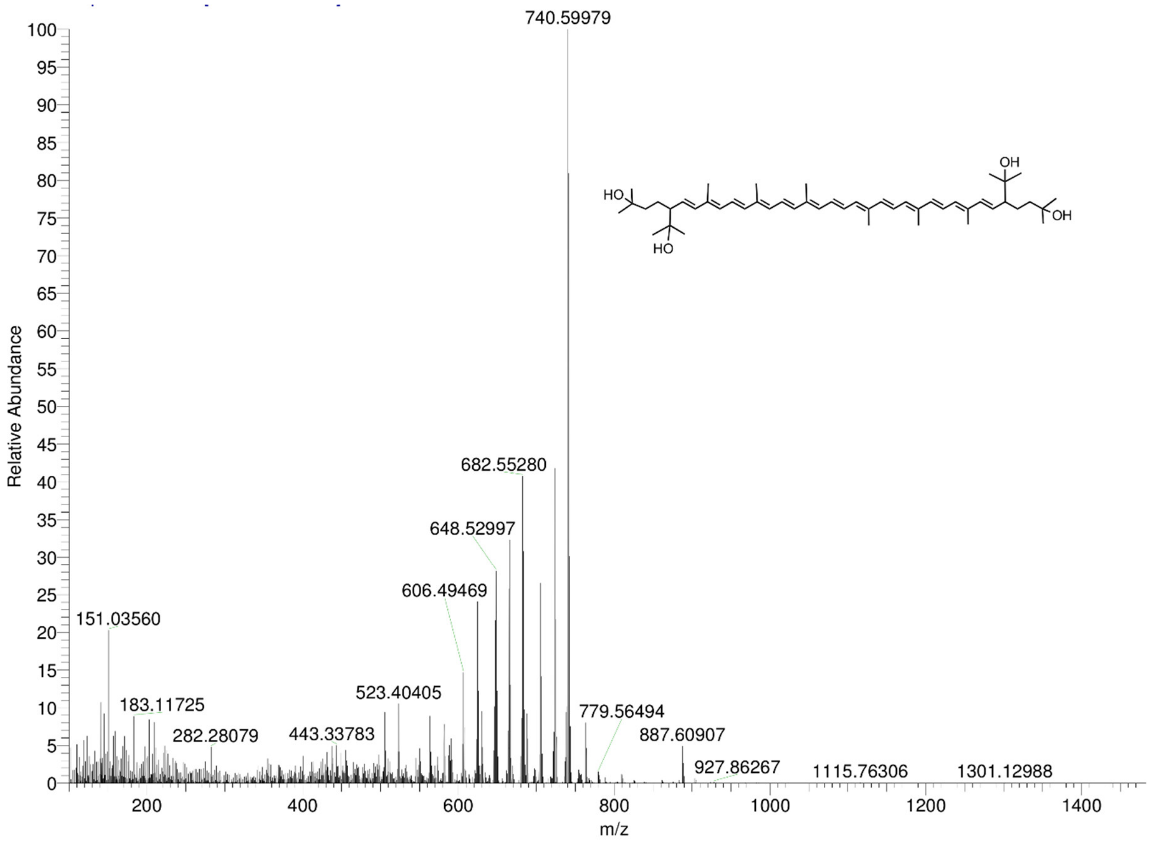

Figure S3. Full MS spectra of Bisanhidrobacterioruberin, peak 17.

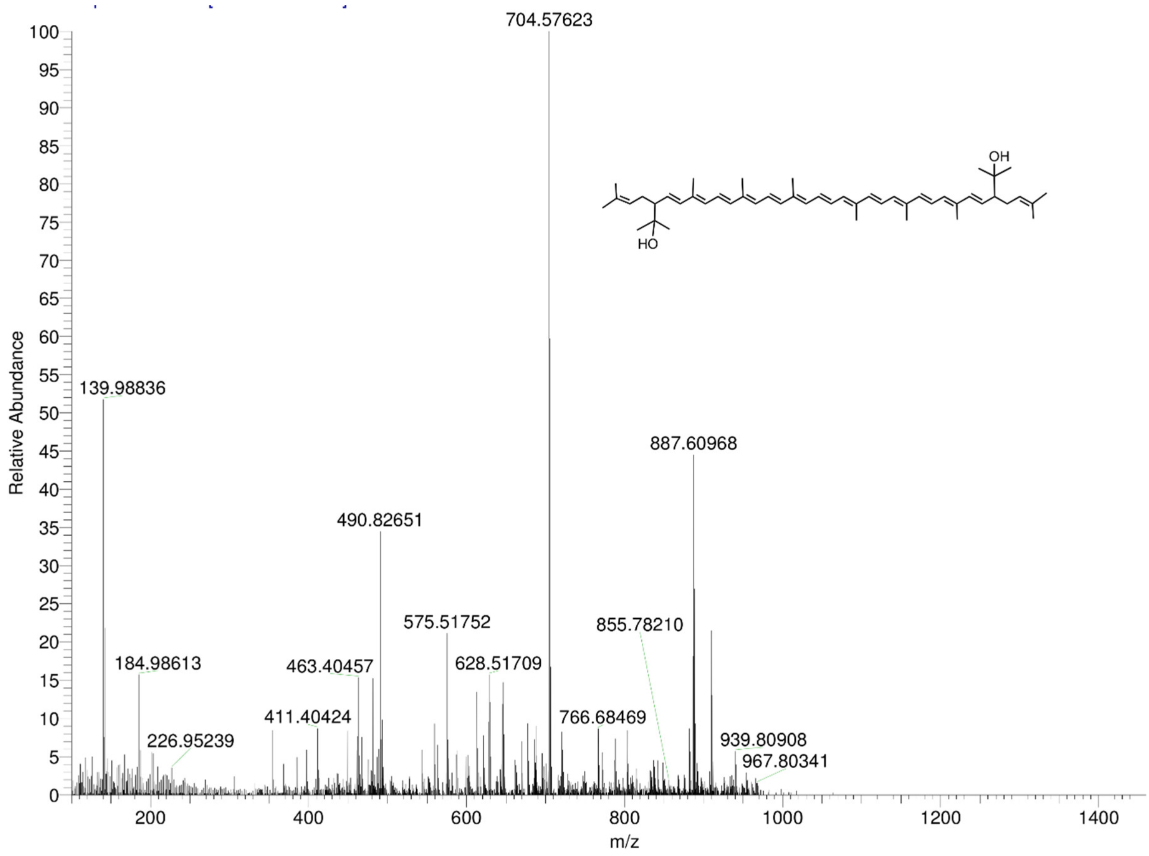

REGISTERED DNA SEQUENCES

LOCUS   MZ576846   1327 bp   DNA   linear   BCT 21-JUL-2021
DEFINITION *Haloarcula* sp. strain TeSe-89 16S ribosomal RNA gene, partial
            sequence.
ACCESSION   MZ576846
VERSION   MZ576846 GI:2069852678
KEYWORDS   .
SOURCE   *Haloarcula* sp.
  ORGANISM *Haloarcula* sp.
            Archaea; Euryarchaeota; Stenosarchaea group; Halobacteria;
            Halobacteriales; Haloarculaceae; Haloarcula; unclassified
            Haloarcula.
REFERENCE   1 (bases 1 to 1327)
  AUTHORS   Lizama-Jimenez,C.
  TITLE   Genus diversity of haloarchaeas in Laguna de Tebenquiche
  JOURNAL   Unpublished
REFERENCE   2 (bases 1 to 1327)
  AUTHORS   Lizama-Jimenez,C., Andrade,D., Campos,V. and Rosello-Mora,R.
  TITLE   Direct Submission
  JOURNAL   Submitted (19-JUL-2021) Tecnologia Medica, Universidad de

Antofagasta, Av. Universidad de Antofagasta, Antofagasta,
Antofagasta 1240000, Chile
COMMENT Sequences were screened for chimeras by the submitter using
Sequencher 4.8.

##Assembly-Data-START##
Sequencing Technology :: Sanger dideoxy sequencing
##Assembly-Data-END##
FEATURES Location/Qualifiers
source 1..1327
/organism="*Haloarcula* sp."
/mol_type="genomic DNA"
/strain="TeSe-89"
/db_xref="taxon:44098"
rRNA <1..>1327
/product="16S ribosomal RNA"
ORIGIN
1 gctagttgca cgagttcaga ctcgtagcat atagctcagt aacacgtggc caaactaccc
61 tacagaccgc ataacctcgg gaaactgagg ccaatagcgg atatcactct cagactggag
121 tgtcgagagt gagaaacgtt ccggcgctgt aggatgtggc tgcggccgat taggtagatg
181 gtggggtaac ggcccaccat gccgataatc ggtacggttg tgagagcaag agccggagac
241 ggtatctgag acaagatacc gggccctacg gggcgcagca ggcgcgaaac ctttacactg
301 cacgacagtg cgataggggg actccgagtg tgagggcata tagccctcgc ttttctgtac
361 cgtaaggtgg tacaggaaca aggactgggc aagaccggtg ccagccgccg cggtaatacc
421 ggcagtccaa gtgatggccg atattattgg gcctaaagcg tccgtagctg ctgtgtaagt
481 ccattgggaa atcgaccagc tcaactggtc ggcgtccggt ggaaactaca cagcttgggg
541 ccgagagact caacgggtac gtccggggta ggagtgaaat cctgtaatcc tggacggacc
601 accaatggga aaccacgttg agagaccgga cccgacagtg agggacgaaa gccagggtct
661 cgaaccggat tagatacccg ggtagtcctg gctgtaaaca atgctcgcta ggtatgtcac
721 gcgccatgag cagtgatgtg ccgtagtgaa gacgataagc gagccgcctg ggaagtacgt
781 ccgcaaggat gaaacttaaa ggaattggcg ggggagcacc acaaccggag gagcctgcgg
841 tttaattgga ctcaacgccg gaaatctcac cggtcccgac agtagtaatg acagtcaggt
901 tgacgacttt actcgacgct actgagagga ggtgcatggc cgccgtcagc tcgtaccgtg
961 aggcgtcctg ttaagtcagg caacgagcga gacccgcact tctagttgcc agcaataccc
1021 ttgaggtagt tgggtacact aggaggactg ccgctgctaa agcggaggaa ggaacgggca
1081 acggtaggtc agtatgcccc gaatggaccg ggcaacacgc gggctacaat ggctctgaca
1141 gtgggatgca acgccgagag gcgaagctaa tctccaaaag gagtcgtagt tcggattgcg
1201 ggctgaaacc cgcccgcatg aagctggatt cggtagtaat cgcgtgtcag aagcgcgcgg
1261 tgaatacgtc cctgctcctt gcacacaccg cccgtcaaag cacccgagtg gggtccggat
1321 gaggccg
//
LOCUS MZ576847 1375 bp DNA linear BCT 21-JUL-2021
DEFINITION *Halorubrum tebenquichense* strain TeSe-86 16S ribosomal RNA gene,
partial sequence.
ACCESSION MZ576847
VERSION MZ576847 GI:2069852679
KEYWORDS .
SOURCE *Halorubrum tebenquichense*
ORGANISM *Halorubrum tebenquichense*
Archaea; Euryarchaeota; Stenosarchaea group; Halobacteria;

Haloferacales; Halorubraceae; Halorubrum.

REFERENCE 1 (bases 1 to 1375)
AUTHORS Lizama-Jimenez,C.
TITLE Genus diversity of haloarchaeas in Laguna de Tebenquiche
JOURNAL Unpublished

REFERENCE 2 (bases 1 to 1375)
AUTHORS Lizama-Jimenez,C., Andrade,D., Campos,V. and Rosello-Mora,R.
TITLE Direct Submission
JOURNAL Submitted (19-JUL-2021) Tecnologia Medica, Universidad de Antofagasta, Av. Universidad de Antofagasta, Antofagasta, Antofagasta 1240000, Chile

COMMENT Sequences were screened for chimeras by the submitter using Sequencher 4.8.

##Assembly-Data-START##
Sequencing Technology :: Sanger dideoxy sequencing
##Assembly-Data-END##

FEATURES Location/Qualifiers
source 1..1375
/organism="*Halorubrum tebenquichense*"
/mol_type="genomic DNA"
/strain="TeSe-86"
/db_xref="taxon:119434"
rRNA <1..>1375
/product="16S ribosomal RNA"

ORIGIN
1 gccattgcta ttgggatccg atttagccat gctagtcgta cgagttcaca ctcgtggcga
61 atagctcagt aacacgtggc caaactaccc ttcggagcgc aataccctcg ggaaactgag
121 gctaatagcg cataccatac caccactgga atgagtggta tgccaaacgc tccggcgccg
181 aaggatgtgg ctgcggccga ttaggtagac ggtggggtaa cggcccaccg tgccaataat
241 cggtatgggt catgagagtg agaacccaga gacggaatct gagacaagat tccgggccct
301 acggggcgca gcaggcgcga aacctttaca ctgcacgaca gtgcgatagg gggatcccaa
361 gtgcacaggc atagcgcctg tgcttttcga caccctaagg cggtgtcaga ataagggctg
421 ggcaagaccg gtgccagccg ccgcggtaat accggcagcc caagtgatgg ccgatcttat
481 tgggcctaaa gcgtccgtag ctggccacgc aagtccatcg ggaaatccac ctgcccaaca
541 ggtgggcgcc cggtagaaac tgcgtggctt gggaccggaa ggcgcgacgg gtacgtccgg
601 ggtaggagtg aaatcccgta atcctggacg gaccgccgat ggcgaaagca cgtcgcgaga
661 acggatccga cagtgaggga cgaaagccag ggtctcgaac cggattagat acccgggtag
721 tcctggccgt aaacaatgtc tgctaggtgt ggctcccact acgagtgggt gctgtgccgt
781 agggaagccg ctaagcagac cgcctgggaa gtacgtccgc aaggatgaaa cttaaaggaa
841 ttggcggggg agcactacaa ccggaggagc ctgcggttta attggactca acgccggaca
901 tctcaccagc atcgactgta gtaatgacga tcaggttgat gaccttatcc gagcttcaga
961 gaggaggtgc atggccgccg tcagctcgta ccgtgaggcg tcctgttaag tcaggcaacg
1021 agcgagaccc gcatccttac ttgccagcag tatcgcgaga tagctgggga cagtagggag
1081 accgccgtgg ctaacacgga ggaaggaacg ggcaacggta ggtcagtatg ccccgaatgt
1141 gctgggcaac acgcgggcta caatggtcga gacaaagggt tccaactccg aaaggagacg
1201 gtaatctcag aaactcgatc gtattcggat tgtgggctgc aactcgccca catgaagctg
1261 gattcggtag taatcgcgtg tcacaagcgc gcggtgaata cgtccctgct ccttgcacac
1321 accgcccgtc aaagcacccg agtgaggtcc ggatgaggcg cgccacgcgc gtcga
//

LOCUS MZ576848 1340 bp DNA linear BCT 21-JUL-2021
DEFINITION *Halorubrum tebenquichense* strain TeSe-85 16S ribosomal RNA gene,
partial sequence.
ACCESSION MZ576848
VERSION MZ576848 GI:2069852680
KEYWORDS .
SOURCE *Halorubrum tebenquichense*
ORGANISM *Halorubrum tebenquichense*
Archaea; Euryarchaeota; Stenosarchaea group; Halobacteria;
Haloferacales; Halorubraceae; Halorubrum.
REFERENCE 1 (bases 1 to 1340)
AUTHORS Lizama-Jimenez,C.
TITLE Genus diversity of haloarchaeas in Laguna de Tebenquiche
JOURNAL Unpublished
REFERENCE 2 (bases 1 to 1340)
AUTHORS Lizama-Jimenez,C., Andrade,D., Campos,V. and Rosello-Mora,R.
TITLE Direct Submission
JOURNAL Submitted (19-JUL-2021) Tecnologia Medica, Universidad de
Antofagasta, Av. Universidad de Antofagasta, Antofagasta,
Antofagasta 1240000, Chile
COMMENT Sequences were screened for chimeras by the submitter using
Sequencher 4.8.

##Assembly-Data-START##
Sequencing Technology :: Sanger dideoxy sequencing
##Assembly-Data-END##
FEATURES Location/Qualifiers
source 1..1340
/organism="*Halorubrum tebenquichense*"
/mol_type="genomic DNA"
/strain="TeSe-85"
/db_xref="taxon:119434"
rRNA <1..>1340
/product="16S ribosomal RNA"
ORIGIN
1 gctagtcgta cgagttcaca ctcgtggcga atagctcagt aacacgtggc caaactaccc
61 ttcggagcgc aataccctcg ggaaactgag gctaatagcg cataccatac caccactgga
121 atgagtggta tgccaaacgc tccggcgccg aaggatgtgg ctgcggccga ttaggtagac
181 ggtggggtaa cggcccaccg tgccaataat cggtatgggt catgagagtg agaacccaga
241 gacggaatct gagacaagat tccgggccct acggggcgca gcaggcgcga aacctttaca
301 ctgcacgaca gtgcgatagg gggatcccaa gtgcacaggc atagcgcctg tgcttttcga
361 caccctaagg cggtgtcaga ataagggctg ggcaagaccg gtgccagccg ccgcggtaat
421 accggcagcc caagtgatgg ccgatcttat tgggcctaaa gcgtccgtag ctggccacgc
481 aagtccatcg ggaaatccac ctgcccaaca ggtgggcgcc cggtagaaac tgcgtggctt
541 gggaccggaa ggcgcgacgg gtacgtccgg ggtaggagtg aaatcccgta atcctggacg
601 gaccgccgat ggcgaaagca cgtcgcgaga acggatccga cagtgaggga cgaaagccag
661 ggtctcgaac cggattagat acccgggtag tcctggccgt aaacaatgtc tgctaggtgt
721 ggctcccact acgagtgggt gctgtgccgt agggaagccg ctaagcagac cgcctgggaa
781 gtacgtccgc aaggatgaaa cttaaaggaa ttggcggggg agcactacaa ccggaggagc
841 ctgcggttta attggactca acgccggaca tctcaccagc atcgactgta gtaatgacga

901 tcaggttgat gaccttatcc gagcttcaga gaggaggtgc atggccgccg tcagctcgta
961 ccgtgaggcg tcctgttaag tcaggcaacg agcgagaccc gcatccttac ttgccagcag
1021 tatcgcgaga tagctgggga cagtagggag accgccgtgg ctaacacgga ggaaggaacg
1081 ggcaacggta ggtcagtatg ccccgaatgt gctgggcaac acgcgggcta caatggtcga
1141 gacaaagggt tccaactccg aaaggagacg gtaatctcag aaactcgatc gtagttcgga
1201 ttgtgggctg caactcgccc acatgaagct ggattcggta gtaatcgcgt gtcacaagcg
1261 cgcggtgaat acgtccctgc tccttgcaca caccgcccgt caaagcaccc gagtgaggtc
1321 cggatgaggc gcgccacgcg
//
LOCUS MZ576849 1342 bp DNA linear BCT 21-JUL-2021
DEFINITION *Haloarcula* sp. strain TeSe-51 16S ribosomal RNA gene, partial
sequence.
ACCESSION MZ576849
VERSION MZ576849 GI:2069852681
KEYWORDS .
SOURCE *Haloarcula* sp.
ORGANISM *Haloarcula* sp.
Archaea; Euryarchaeota; Stenosarchaea group; Halobacteria;
Halobacteriales; Haloarculaceae; *Haloarcula*; unclassified
Haloarcula.
REFERENCE 1 (bases 1 to 1342)
AUTHORS Lizama-Jimenez,C.
TITLE Genus diversity of haloarchaeas in Laguna de Tebenquiche
JOURNAL Unpublished
REFERENCE 2 (bases 1 to 1342)
AUTHORS Lizama-Jimenez,C., Andrade,D., Campos,V. and Rosello-Mora,R.
TITLE Direct Submission
JOURNAL Submitted (19-JUL-2021) Tecnologia Medica, Universidad de
Antofagasta, Av. Universidad de Antofagasta, Antofagasta,
Antofagasta 1240000, Chile
COMMENT Sequences were screened for chimeras by the submitter using
Sequencher 4.8.

##Assembly-Data-START##
Sequencing Technology :: Sanger dideoxy sequencing
##Assembly-Data-END##
FEATURES Location/Qualifiers
source 1..1342
/organism="*Haloarcula* sp."
/mol_type="genomic DNA"
/strain="TeSe-51"
/db_xref="taxon:44098"
rRNA <1..>1342
/product="16S ribosomal RNA"
ORIGIN
1 gctagttgca cgagttcaga ctcgtagcat atagctcagt aacacgtggc caaactaccc
61 tacagaccgc gataacctcg ggaaactgag gccaatagcg gatataactc tcaggctgga
121 gtgccgagag ttagaaacgt tccggcgctg taggatgtgg ctgcggccga ttaggtagat
181 ggtggggtaa cggcccacca tgccgataat cggtacaggt tgtgagagca agaccggaga
241 cggtatctga gacaagatac cgggccctac ggggcgcagc aggcgcgaaa cctttacact

301 gcacgacagt gcgatagggg gactccgagt gtgagggcat atagccctcg cttttctgta
361 ccgtaaggtg gttcaggaac aaggactggg caagaccggt gccagccgcc gcggtaatac
421 cggcagtcca agtgatggcc gatattattg ggcctaaagc gtccgtagct gctggaagtc
481 cattgggaaa tcgaccagct caactggtcg gcgtccggtg gaaactcaca gcttggggcc
541 gagagactca cgggtacgtc cggggtagga gtgaaatcct gtaatcctgg acggaccacc
601 aatggggaaa ccacgtgaga gacggacccg acagtgaggg acgaaagcca gggtctcgaa
661 ccggattaga tacccgggta gtcctggctg taaacaatgc tcgctaggtt gtcaccgcca
721 tgagcagtga tgtgccgtag tgaagacgat aagcgagccg cctgggaagt acgtccgcaa
781 ggatgaaact taaaggaatt ggcgggggag caccacaacc ggaggagcct gcggtttaat
841 tggactcaac gccggaaatc tcaccggtcc cgacagtagt aatgacagtc aggttgacga
901 ctttactcga cgctactgag aggaggtgca tggccgccgt cagctcgtac cgtgaggcgt
961 cctgttaagt caggcaacga gcgagacccg cacttctagt tgccagcaat acccttgagg
1021 tagttgggta cactaggagg actgccgctg ctaaagcgga ggaaggaacg ggcaacggta
1081 ggtcagtatg ccccgaatgg accgggcaac acgcgggcta caatggctct gacagtggga
1141 tgcaacgccg agaggcgaag ctaatctcca aacggagtcg tagttcggat tgcgggctga
1201 aacccgcccg catgaagctg gattcggtag taatcgcgtg tcagaagcgc gcggtgaata
1261 cgtccctgct ccttgcacac accgcccgtc aaagcacccg agtggggtcc ggatgaggcc
1321 gtcatgcgac ggtcgaatct gg
//
LOCUS MZ576850 1316 bp DNA linear BCT 21-JUL-2021
DEFINITION *Haloarcula* sp. strain TeSe-41 16S ribosomal RNA gene, partial
sequence.
ACCESSION MZ576850
VERSION MZ576850 GI:2069852682
KEYWORDS .
SOURCE *Haloarcula* sp.
ORGANISM *Haloarcula* sp.
Archaea; Euryarchaeota; Stenosarchaea group; Halobacteria;
Halobacteriales; Haloarculaceae; Haloarcula; unclassified
Haloarcula.
REFERENCE 1 (bases 1 to 1316)
AUTHORS Lizama-Jimenez,C.
TITLE Genus diversity of haloarchaeas in Laguna de Tebenquiche
JOURNAL Unpublished
REFERENCE 2 (bases 1 to 1316)
AUTHORS Lizama-Jimenez,C., Andrade,D., Campos,V. and Rosello-Mora,R.
TITLE Direct Submission
JOURNAL Submitted (19-JUL-2021) Tecnologia Medica, Universidad de
Antofagasta, Av. Universidad de Antofagasta, Antofagasta,
Antofagasta 1240000, Chile
COMMENT Sequences were screened for chimeras by the submitter using
Sequencher 4.8.

##Assembly-Data-START##
Sequencing Technology :: Sanger dideoxy sequencing
##Assembly-Data-END##
FEATURES Location/Qualifiers
source 1..1316
/organism="*Haloarcula* sp."
/mol_type="genomic DNA"

/strain="TeSe-41"
/db_xref="taxon:44098"
rRNA <1..>1316
/product="16S ribosomal RNA"
ORIGIN
1 gttgcacgag ttcagactcg tagcatatag ctcagtaaca cgtggccaaa ctaccctaca
61 gaccgcgata acctcgggaa actgaggcca atagcggata taactctcag gctggagtgc
121 cgagagttag aaacgttccg gcgctgtagg atgtggctgc ggccgattag gtagatggtg
181 gggtaacggc ccaccatgcc gataatcggt acgggttgtg agagcaagac ccggagacgg
241 tatctgagac aagataccgg gccctacggg gcgcagcagg cgcgaaacct ttacactgca
301 cgacagtgcg atagggggac tccgagtgtg agggcatata gccctcgctt ttctgtaccg
361 taaggtggtt caggaacaag gactgggcaa gaccggtgcc agccgccgcg gtaataccgg
421 cagtccaagt gatggccgat attattgggc ctaaagcgtc cgtagcgctg aagtccattg
481 ggaaatcgac cgctcaacgg tcggcgtccg gtggaaactc agcttggggc cgagactcac
541 gggtacgtcc ggggtaggag tgaaatcctg taatcctgga cggaccacca atggggaaac
601 cacgtgagac ggacccgacg tgagggacga aagccagggt ctcgaaccgg attagatacc
661 cgggtagtcc tggctgtaaa cgatgctcgc taggtgtgtc cgccatgagc tggatggccg
721 tagtgaagcg ataagcgagc cgcctgggaa gtacgtcgca aggatgaaac ttaaaggaat
781 tggcggggga gcaccacaac cggaggagcc tgcggtttaa ttggactcaa cgccggaaat
841 ctcaccggtc ccgacagtag taatgacagt caggttgacg actttactcg acgctactga
901 gaggaggtgc atggccgccg tcagctcgta ccgtgaggcg tcctgttaag tcaggcaacg
961 agcgagaccc gcacttctag ttgccagcaa taccсttgag gtagttgggt acactaggag
1021 gactgccgct gctaaagcgg aggaaggaac gggcaacggt aggtcagtat gccccgaatg
1081 gaccgggcaa cacgcgggct acaatggctc tgacagtggg atgcaacgcc gagaggcgac
1141 gctaatctcc aaacggagtc gtagttcgga ttgcgggctg aaacccgccc gcatgaagct
1201 ggattcggta gtaatcgcgt gtcagaagcg cgcggtgaat acgtccctgc tccttgcaca
1261 caccgcccgt caaagcaccc gagtggggtc cggatgaggc cgtcatgcga cggtcg
//
LOCUS MZ576851 1362 bp DNA linear BCT 21-JUL-2021
DEFINITION *Haloarcula* sp. strain ALT-23 16S ribosomal RNA gene, partial
sequence.
ACCESSION MZ576851
VERSION MZ576851 GI:2069852683
KEYWORDS .
SOURCE *Haloarcula* sp.
ORGANISM *Haloarcula* sp.
Archaea; Euryarchaeota; Stenosarchaea group; Halobacteria;
Halobacteriales; Haloarculaceae; Haloarcula; unclassified
Haloarcula.
REFERENCE 1 (bases 1 to 1362)
AUTHORS Lizama-Jimenez,C.
TITLE Genus diversity of haloarchaeas in Laguna de Tebenquiche
JOURNAL Unpublished
REFERENCE 2 (bases 1 to 1362)
AUTHORS Lizama-Jimenez,C., Andrade,D., Campos,V. and Rosello-Mora,R.
TITLE Direct Submission
JOURNAL Submitted (19-JUL-2021) Tecnologia Medica, Universidad de
Antofagasta, Av. Universidad de Antofagasta, Antofagasta,
Antofagasta 1240000, Chile
COMMENT Sequences were screened for chimeras by the submitter using

```
            Sequencher 4.8.

            ##Assembly-Data-START##
            Sequencing Technology :: Sanger dideoxy sequencing
            ##Assembly-Data-END##
FEATURES             Location/Qualifiers
     source          1..1362
                     /organism="Haloarcula sp."
                     /mol_type="genomic DNA"
                     /strain="ALT-23"
                     /db_xref="taxon:44098"
     rRNA            <1..>1362
                     /product="16S ribosomal RNA"
ORIGIN
        1 gccattgcta tcggagtccg atttagccat gctagttgca cgagttcaga ctcgtaggca
       61 tatagctcag taacacgtgg ccaaactacc ctacagaccg cataacctcg ggaaactgag
      121 gccaatagcg gatataactc tcaggctgga gtgcgagagt tagaaacgtt ccggcgctgt
      181 aggatgtggc tgcggccgat taggtagatg gtggggtaac ggcccaccat gccgataatc
      241 ggtacaggtt gtgagagcaa gagcctggag acggtatctg agacaagata ccgggcccta
      301 cggggcgcag caggcgcgaa acctttacac tgcacgacag tgcgataggg ggactccgag
      361 tgtgagggca tatagccctc gcttttctgt accgtaaggt ggtacaggaa caaggactgg
      421 gcaagaccgg tgccagccgc cgcggtaata ccggcagtcc aagtgatggc cgatattatt
      481 gggcctaaag cgtccgtagc tgctgtgtaa gtccattggg aaatcgacca gctcaactgg
      541 tcggcgtccg gtggaaacta cacagcttgg ggccgagaga ctcggggtac gtccggggta
      601 ggagtgaaat cctgtaatcc tggacggacc accaatgggg aaaccacgtt agagaccgga
      661 cccgacagtg agggacgaaa gccagggtct cgaaccggat tagatacccg ggtagtcctg
      721 gctgtaaaca atgctcgcta ggtatgtcac gcgccatgag cacgtgatgt gccgtagtga
      781 agacgataag cgagccgcct gggaagtacg tccgcaagga tgaaacttaa aggaattggc
      841 gggggagcac cacaaccgga ggagcctgcg gtttaattgg actcaacgcc ggaaatctca
      901 ccggtcccga cagtagtaat gacagtcagg ttgacgactt tactcgacgc tactgagagg
      961 aggtgcatgg ccgccgtcag ctcgtaccgt gaggcgtcct gttaagtcag gcaacgagcg
     1021 agacccacac ttctagttgc cagcaatacc cttgaggtgt tgggtacact aggaggactg
     1081 ccgctgctaa agcggaggaa ggaagggcaa cggtaggtca gtatgccccg aatggaccgg
     1141 gcaacacgcg ggctacaatg gctctgacag tgggatgcac gccgagaggc gaagctaatc
     1201 tccaaacgga gtcgtattcg gattgcgggc tgaaacccgc ccgcatgaag ctggattcgg
     1261 tagtaatcgc gtgtcagaag cgcgcggtga atacgtccct gctcttgcac acaccgcccg
     1321 tcaaagcacc cgagtggggt ccggatgagg ccgtcatgcg ac
//
```
